# Constructive adaptation of 3D-printable polymers in response to typically destructive aquatic environments

**DOI:** 10.1093/pnasnexus/pgac139

**Published:** 2022-07-29

**Authors:** Kunhao Yu, Zhangzhengrong Feng, Haixu Du, Kyung Hoon Lee, Ketian Li, Yanchu Zhang, Sami F Masri, Qiming Wang

**Affiliations:** Sonny Astani Department of Civil and Environmental Engineering, University of Southern California, Los Angeles, CA 90089, USA; Sonny Astani Department of Civil and Environmental Engineering, University of Southern California, Los Angeles, CA 90089, USA; Sonny Astani Department of Civil and Environmental Engineering, University of Southern California, Los Angeles, CA 90089, USA; Sonny Astani Department of Civil and Environmental Engineering, University of Southern California, Los Angeles, CA 90089, USA; Sonny Astani Department of Civil and Environmental Engineering, University of Southern California, Los Angeles, CA 90089, USA; Sonny Astani Department of Civil and Environmental Engineering, University of Southern California, Los Angeles, CA 90089, USA; Sonny Astani Department of Civil and Environmental Engineering, University of Southern California, Los Angeles, CA 90089, USA; Sonny Astani Department of Civil and Environmental Engineering, University of Southern California, Los Angeles, CA 90089, USA

**Keywords:** constructive adaptation, self-strengthening, self-healing, 3D-printing, bio-inspiration

## Abstract

In response to environmental stressors, biological systems exhibit extraordinary adaptive capacity by turning destructive environmental stressors into constructive factors; however, the traditional engineering materials weaken and fail. Take the response of polymers to an aquatic environment as an example: Water molecules typically compromise the mechanical properties of the polymer network in the bulk and on the interface through swelling and lubrication, respectively. Here, we report a class of 3D-printable synthetic polymers that constructively strengthen their bulk and interfacial mechanical properties in response to the aquatic environment. The mechanism relies on a water-assisted additional cross-linking reaction in the polymer matrix and on the interface. As such, the typically destructive water can constructively enhance the polymer’s bulk mechanical properties such as stiffness, tensile strength, and fracture toughness by factors of 746% to 790%, and the interfacial bonding by a factor of 1,000%. We show that the invented polymers can be used for soft robotics that self-strengthen matrix and self-heal cracks after training in water and water-healable packaging materials for flexible electronics. This work opens the door for the design of synthetic materials to imitate the constructive adaptation of biological systems in response to environmental stressors, for applications such as artificial muscles, soft robotics, and flexible electronics.

Significance Statement Turning destructive environmental stressors into constructive factors is a golden law of adaptation and survival for biological systems. However, such a law is typically absent in synthetic engineering systems because synthetic materials generally respond destructively with failures to destructive environmental stressors. In this report, we take the aquatic stressor as an example to apply the biological law of adaptation and survival to synthetic materials. We show that synthetic polymers with smart chemical designs can behave like a biological system to turn the destructive aquatic stressor into constructive factors to drastically enhance bulk and interfacial mechanical properties.

## Introduction

**Fig. 1.**
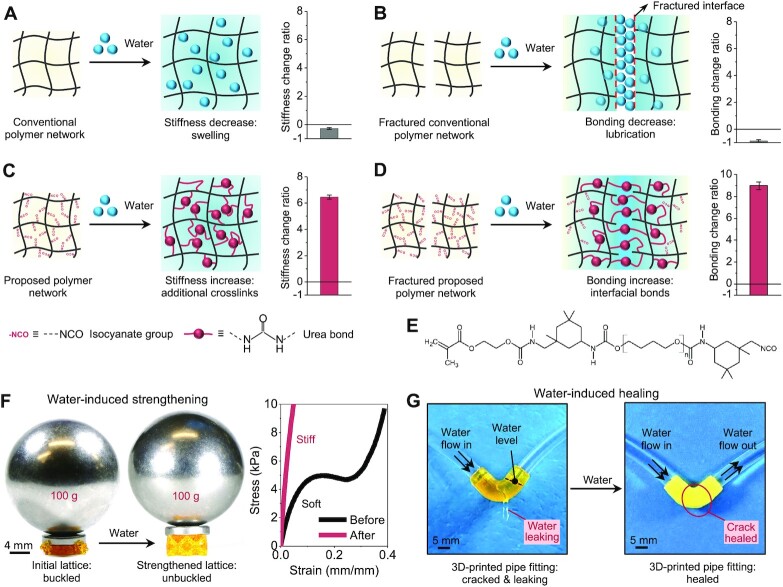
The overall mechanism of constructive adaptation in the aquatic environment. (A) Schematic illustration of water-swelling–induced stiffness decrease of a conventional bulk polymer network and the corresponding stiffness change ratio of a conventional polyurethane polymer after being immersed in the water for 24 h. The stiffness change ratio is calculated as $( {{E_w} - {E_i}} )/{E_i}$, where ${E_i}$ is Young’s modulus of the initial sample and ${E_w}$ is Young’s modulus of the sample after water immersion. (B) Schematic illustration of water-induced bonding decrease of a fractured conventional polymer network and the corresponding bonding change ratio of a fractured conventional polyurethane polymer after being immersed in the water for 24 h. The bonding change ratio is calculated as $( {{B_w} - {B_i}} )/{B_i}$, where ${B_i}$ is the bonding force of a fractured polymer put into contact immediately after damaged and ${B_w}$ is the bonding force of a fractured polymer after water immersion. (C) Schematic illustration of water-induced stiffness increase of the proposed polymer network and the corresponding stiffness change ratio after being immersed in the water for 24 h (data from Fig. 2F). (D) Schematic illustration of water-induced bonding increase of a fractured proposed polymer and the corresponding bonding change ratio after being treated with water (data from Fig. 3G). (E) Chemical structure of the polymer resin that features both acrylate and isocyanate distal groups. (F) Water-induced strengthening of a 3D-printed Octet lattice structure (0.11 g) that is loaded with a 100-g metal ball, and the corresponding compressive stress–strain curves of the structure before and after being immersed in the water for 24 h. (G) Water-induced crack healing of a 3D-printed pipe fitting (details in Fig. S6).

Although engineering and biological systems are surrounded by similar destructive environmental stressors, such as load imposition, light exposure, and water immersion, their responses are typically different. Biological systems exhibit an extraordinary adaptive capacity by turning destructive environmental stressors into constructive factors. For example, bone and muscle turn the typically destructive mechanical loads into constructive factors to build mass and mechanical strength (1–6). Plants harness sunlight, which otherwise degrades substance (7), to constructively synthesize polysaccharides and grow stiffness and strength (8, 9). Engineering systems, on the contrary, typically do not possess the intelligence of constructive adaptation but weaken in response to destructive environmental stressors. Take the response of polymers to the aquatic environment as an example: Water molecules typically compromise the mechanical properties of the polymer network in the bulk and on the interface. When water molecules migrate into a bulk polymer network, the polymer network swells, and its stiffness and strength reduce (Fig. 1A) (10–12). When water molecules exist on the polymer interface, they lubricate the interface and reduce the interfacial adhesion or bonding (Fig. 1B) (13, 14). Such destructive responses may drastically limit the use of synthetic polymers in the fields that require the stability of bulk and interfacial mechanical properties in the aquatic environment.

Inspired by the biological systems, we here report a class of 3D-printable synthetic polymers that constructively strengthen their bulk and interfacial mechanical properties in response to the typically destructive aquatic environment (Fig. 1C and D). The mechanism relies on a water-assisted additional cross-linking reaction in the polymer matrix and on the interface, thus overcoming the property compromise due to swelling and lubrication. The proposed mechanism can constructively enhance the bulk mechanical properties such as stiffness, tensile strength, and fracture toughness by factors of 746% to 790% (Fig. 1C), and the interfacial bonding by a factor of 1,000% (Fig. 1D). The proposed polymer is molecularly designed to be photocurable, thus facilitating 3D printing of various complex structures via a stereolithography system. To highlight the impact of the constructive adaptation, we show a 3D-printed robotic arm can strengthen its lifting capability after training in the water, though it weakens after training in the air. We also demonstrate a robotic fish with a 3D-printed polymer fin can swim faster and heal cracks after training in the water. Moreover, the proposed polymer can be used as a water-healable packaging material that can efficiently resist water-induced performance degradation of flexible electronics. The paradigm in this work provides a unique platform for innovating bioinspired constructive adaptation of engineering materials in response to typically destructive aquatic environments.

## Results

### The overall mechanism of constructive adaptation in the aquatic environment

We have designed a class of 3D-printable polymers that incorporate active chemical groups to constructively strengthen the material matrix and interface in the aquatic environment (Fig. 1C and D). To synthesize the polymer, we molecularly design a polymer resin that features both acrylate and isocyanate distal groups (NCO groups) (Fig. 1E and Fig. S1). The acrylate groups can facilitate the photoradical-initiated addition reaction for diverse photopolymerization-based 3D-printing processes (such as stereolithography, Fig. S2) (15–17). After photopolymerization, the NCO groups become tails on side chains within the polymer matrix (Fig. S3). One water molecule can bridge two side chains by reacting with two NCO groups to form urethane bonds (Fig. S4) (18–20). Within the polymer matrix, such a chemical bridging mechanism enables the formation of new cross-links, additional to the cross-linking during the photopolymerization process (Fig. 1C). The additional cross-links within the matrix can constructively enhance the material stiffness and strength (3–5, 9), thus offsetting the stiffness reduction effect due to the water-induced polymer swelling. Around the polymer interface, the chemical bridging can also constitute relatively strong interfacial bonding, thus healing the interfacial cracks (Fig. 1D). Note that the chemical structure of the proposed polymer is different from that of the traditional moisture-cured polyurethane (21). Because the proposed polymer after the reaction with water is essentially a double-network polymer: The first one formed via photopolymerization and the second one formed via water–NCO reaction; however, the moisture-cured polyurethane only contains the second network.

As a quick demonstration of the concept, we use the proposed polymer to 3D-print a lattice structure that drastically increases the weight-sustaining capability after being immersed in the water for 24 h (Fig. 1F and Fig. S5). The strengthened lattice structure is able to sustain a weight of 100 g that is around 900 times its own weight without buckling beams, while the unstrengthened lattice buckled. As another example, a broken 3D-printed pipe fitting can heal the crack and resume water flow after being immersed in the water for 24 h (Fig. 1G and Fig. S6, Movie S1).

### Water-induced bulk strengthening

**Fig. 2.**
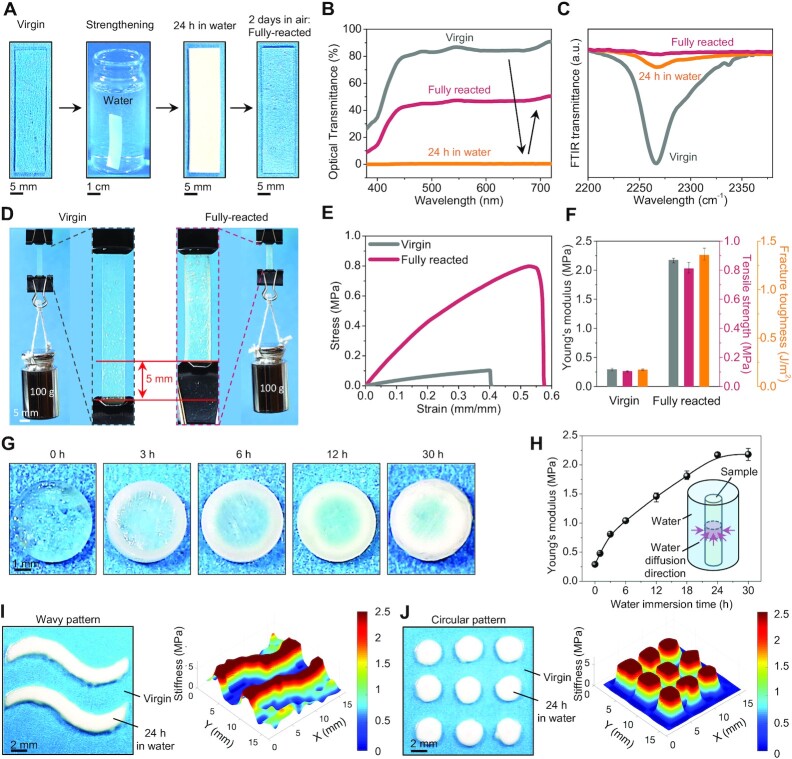
Water-induced bulk strengthening. (A) Image sequence of a strip sample at the virgin, strengthening, 24 h in water, and fully reacted states. (B) Optical transmittance spectra of the strip sample at different states. (C) FTIR spectra within the range of 2,200 $c{m^{ - 1}}$ to 2,375 $c{m^{ - 1}}$ of the sample at different states. (D) A strip sample at the virgin and fully reacted states loaded with a weight of 100 g. The zoom-in images show the length difference of the sample at two states. (E) Uniaxial tensile stress–strain curves of the sample at virgin and fully reacted states. (F) Young’s moduli, tensile strengths, and fracture toughnesses of the sample at virgin and fully reacted states. (G) Cross-section images of a cylindrical sample immersed in water for various periods of time. (H) The longitudinal Young’s modulus of the cylindrical sample as a function of the water-immersion time. The inset illustrates the water diffusion direction. (I, J) Water-induced local strengthening of a plate sample with wavy and circular patterns of water supplies, and the corresponding stiffness mapping of the strengthened samples. Error bars in (F) and (H) represent standard deviations over three to five samples.

To systematically characterize the water-induced bulk strengthening, we first investigate different stages of the strengthening process with a sample strip (Fig. 2A). When the sample is fabricated (the virgin state, thickness ∼1 mm), the sample is nearly transparent with an optical transmittance of around 85% within 450 to 720 nm (Fig. 2A and B). Once being immersed in the water for 24 h, the sample’s color gradually turns opaque and milky with an optical transmittance of nearly 0% within the whole visible light range (400 to 720 nm). Such color change is primarily due to the water diffusion into the polymer matrix and its reaction with the NCO groups. The swelling ratio of the sample is around 11% after being immersed in water for 24 h and remains at the same level after being immersed for 7 days (Fig. S7). After being taken out of the water, the sample is resting in the air for 2 days to allow full reaction between the diffused water molecules and the NCO groups within the matrix, and then the sample color becomes semitransparent with an optical transmittance of around 50% within 450 to 720 nm. To examine the concentration evolution of the NCO groups in the polymer matrix, we employ Fourier transform infrared (FTIR) spectrometer to measure the transmittance of the sample around 2,270 $c{m^{ - 1}}$ that is corresponding to the NCO bond stretching vibration (9, 22). A distinct peak centered at 2,270 $c{m^{ - 1}}$ is observed in the virgin state (Fig. 2C and Fig. S8). After the water immersion for 24 h, the peak dramatically drops, indicating that most of the NCO groups have been consumed by water molecules. After full reaction in the air for 2 days, the FTIR peak disappears, revealing that all the NCO groups are fully reacted in the material matrix. We quantify the concentration of the NCO groups by calculating the area under the distinct peak and normalizing the area with the peak area at the virgin state (Fig. S9). The concentration of the NCO groups at the fully reacted state has a concentration of less than 1%. We further investigate the mechanical properties of the sample in the virgin and fully reacted state. When loaded with a weight of 100 g, the virgin sample is stretched 40% more compared to the fully reacted sample (i.e. strengthened sample), indicating higher stiffness of the fully reacted sample (Fig. 2D). Under uniaxial tensile tests, the fully reacted sample exhibits higher Young’s modulus and tensile strength than those of the virgin sample by factors of 746% and 784%, respectively (Figs. 2E and F and Fig. S10). We also employ pure-shear fracture tests to measure the fracture toughnesses (Fig. S11) and find that the fracture toughness of the fully reacted sample is 7.9 times that of the virgin sample (Fig. 2F). Our results imply that the water-induced formation of additional cross-links not only stiffens the polymer network but also enhances the material capability in resisting crack propagation.

We hypothesize that the water–NCO reaction within the material bulk is facilitated by the water diffusion through the polymer matrix. To test this hypothesis, we image the cross-section of cylindrical samples (the length of 20 mm much larger than the diameter of 5 mm) after being immersed in the water for various periods of time. From the cross-section images, the water molecules gradually migrate toward the center of the sample as the immersion time increases (Fig. 2G). After various water-immersion periods, we take out the samples, allow the full reaction in the air for 2 days, and then measure the stiffness of the cylindrical samples along the longitudinal direction. We find that the stiffnesses of the cylindrical samples increase with increasing immersion periods until reaching a plateau after 24 h (Fig. 2H). Higher temperature of the water can accelerate the stiffness-increasing process (Fig. S12). The results in Fig. 2G and H not only verify that the water-induced material strengthening is facilitated and limited by water diffusion, but also confirm that the water molecules can thoroughly react with the sample strip (thickness ∼1 mm) in Fig. 2A to F. The mechanism of diffusion-limited material strengthening allows us to harness confined water to enable local strengthening (Fig. 2I and J and Fig. S13). With patterned water supplies on a plate sample, only the locations in direct contact with water for 24 h turn milky (Fig. 2I and J). After full reaction in the air for 2 days, indentation tests reveal that the stiffnesses of the reacted regions are around 7 times those of the nonreacted regions (Fig. 2I and J).

### Water-induced interfacial healing and bonding

**Fig. 3.**
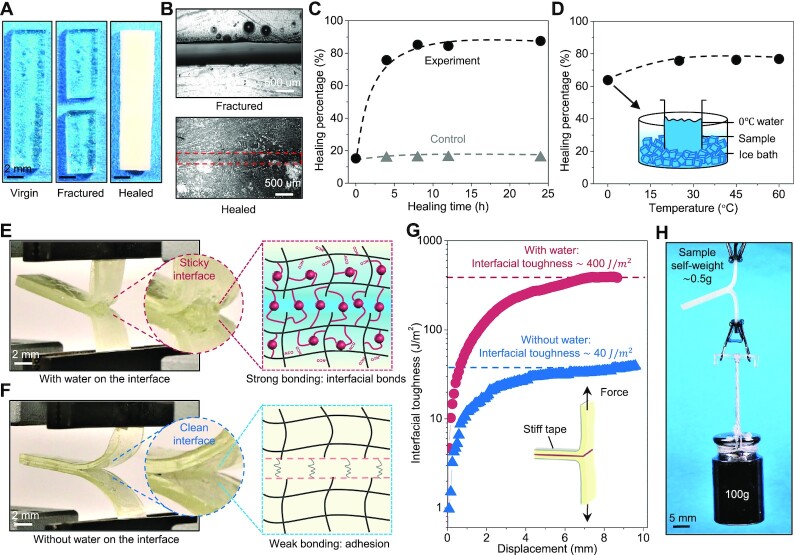
Water-induced interfacial healing and bonding. (A) A strip sample at the the virgin, fractured, and healed states. The sample is cut into two parts with a blade, brought into contact, and then immersed in the water for 8 h to heal. (B) Microscope images of the crack interface at fractured and healed states. (C) Healing percentages of the experimental (with water) and control (without water) groups for various healing time. The healing percentage is defined as the tensile strength of the healed sample normalized by that of the virgin sample with the same water-immersion time. (D) The healing percentage of the samples healed at different water temperatures for 4 h. The inset schematic shows the experimental setup of the sample healing at an ice-water bath of $0^\circ C$. (E) $180^\circ $ peeling test of two sample plates stacked with water spray in between for 2 days. The inset illustrates the sticky interface during the peeling test. (F) $180^\circ $ peeling test of two sample plates stacked without water spray for 2 days. The inset illustrates the clean interface during the peeling test. (H) The interfacial toughness of the experimental and control samples during the $180^\circ $ peeling test. The interfacial toughness is calculated as $2F/w$, where *F* is the loading and *w* is the width of the tested samples. (G) Two sample plates (0.5 g) stacked with a strong water-induced bonding loaded by a weight of 100 g.

The water–NCO reaction around two interfaces can facilitate the interfacial healing of a fractured polymer (Fig. 1D). To demonstrate this concept, we cut a sample strip into two parts and then bring back into contact, followed by immersing the sample in the water (Fig. 3A). After water immersion for 8 h, the fractured interface can be nicely healed, verified by the smooth interface shown in the microscopic images (Fig. 3A and B). To quantify the healing performance, we uniaxially stretch the healed sample with various water-immersion periods until rupture, and then calculate the healing percentage by normalizing the healed tensile strength over the tensile strength of the unfractured sample immersed in water for the same period. We find that the healing percentage increases with increasing water-immersion time until reaching a plateau of around 85% after 8 h (Fig. 3C, black data points). As control experiments, we cut samples into two parts and then bring them back into contact, followed by resting in the air for various periods of time. The control experiments reveal that the interfacial bonding remains below 20% of the virgin tensile strength, without increasing over 25 h (Fig. 3C, gray data points). It is noted that the bonding at the water-immersion time of 0 h shown in Fig. 3C represents the material adhesion of the fractured surface. In addition, we find that the water-induced interfacial healing performance is not drastically compromised by lowering the temperature to 0°C. The healing percentage for the water immersion of 4 h at 25°C is around 75%, while it only slightly reduces to 65% when the water temperature changes to 0°C (Fig. 3D). This may be because the reaction rate between water molecules and NCO groups is only slightly compromised when the temperature reduces from 25°C to 0°C (18–20).

Not only assisting healing of cracked interfaces, the water–NCO reaction can also assist the interfacial bonding of two bulk polymers. To evaluate the water-induced interfacial bonding, we spray water on the interfaces of two rectangular samples, stack two samples together for 2 days, and then conduct a $180^\circ $ peeling test to measure the interfacial toughness (Fig. 3E). We find that the water-treated samples can establish a strong bonding with a sticky interface during the peeling test, indicating new bonding formed between two materials (Fig. 3E). In contrast, the samples that stack together without spraying water on the contacting interface exhibit a clean interface during the peeling test (Fig. 3F). Such a sharp difference in the peeling interface is because that the former strong bonding is primarily attributed to water-induced newly formed chemical bridging on the interface, while the latter weak bonding is merely due to the polymer adhesion. Quantitatively, the interfacial toughness of the bonded samples with the water spraying is around 400 ${\rm{J\,\,}}{{\rm{m}}^{ - 2}}$, which is 10 times that without the water spraying (Fig. 3G). Interestingly, the water-induced bonding between two bulk samples shows the capability of hanging a weight that is more than 200 times the samples’ own weight (Fig. 3H).

### Constructive strengthening of robotic arms in the aquatic environment

**Fig. 4.**
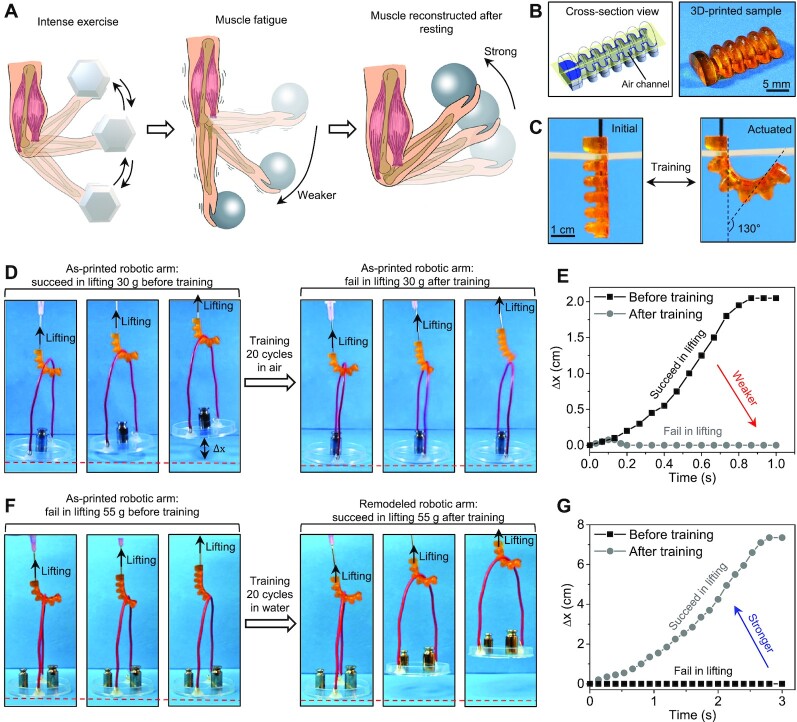
Constructive strengthening of robotic arms in the aquatic environment. (A) Schematics of arm training, fatigue, and reconstruction process. (B) Cross-section view of the computer-aided design model and the 3D-printed pneumatic robotic arm. (C) Reversible actuation of the 3D-printed robot arm. (D) Image sequence to show the actuation of the as-printed robotic arm for lifting a weight of 30 g before and after the training in the air for 20 cycles (followed by resting in the air for 24 h). (E) Displacement and time history of the weight in (D). (F) Image sequence to show the actuation of the as-printed robotic arm for lifting a weight of 55 g before and after the training in the water for 20 cycles (followed by resting in water for 24 h). (G) Displacement and time history of the weight in (F).

Next, we show that the constructive adaptation of the new polymer in the aquatic environment can nicely mimic the constructive training of the human muscle (Fig. 4). Human muscle after training may experience fatigue by partially damaging the muscle microstructures, leading to a capability compromise in exerting a lifting force (Fig. 4A). The fatigued muscle can be restored and strengthened by resting to reconstruct and remodel its microstructures, thus being stronger to exert a larger lifting force (1–5). Here, we try to mimic the constructive training of the human muscle with a 3D-printed robotic arm (Fig. 4B). The 3D-printed robotic arm can be pneumatically actuated to reversibly bend by 130° (Fig. 4C). Since the bending angle is larger than 90°, we expect the actuated robotic arm to imitate the real arm to lift weights. In the first example shown in Fig. 4D, the as-printed robotic arm can successfully lift a weight of 30 g in its first pneumatic actuation; however, after 20 cycles of actuation in the air (4 s cycle^−1^) followed by resting in the air for 24 h, the robotic arm fails in lifting the same weight of 30 g (Fig. 4D and E and Movie S2). The performance degradation after training in the air is because that the continuous actuation of the robotic arm causes the fatigue and stress-softening of the polymer by damaging the polymer microstructure during the actuation (23). The stress-softening behavior is verified by cyclic loading tests shown in Fig. S14. In the second example shown in Fig. 4F, the as-printed robotic arm fails in lifting a weight of 55 g that is over the arm’s initial carrying capability. After being trained for 20 cycles in water (4 s cycle^−1^) followed by resting in water for 24 h, the trained robotic arm can successfully lift the weight of 55 g (Fig. 4F and G and Movie S3). The performance improvement after training in water reveals that the water-induced material strengthening can constructively overcome the cyclic-load–induced material fatigue.

### Strengthening and healing of robotic fish fin in the aquatic environment

**Fig. 5.**
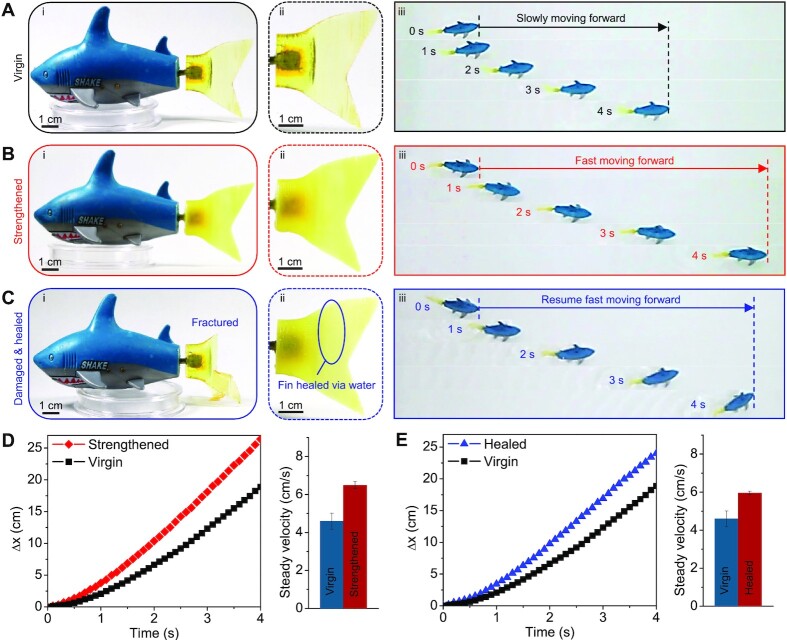
Strengthening and healing of robotic fish fins. (A) (i) A 3D-printed fish fin at the virgin state installed on a robotic fish. (ii) Zoom-in view of the fish fin at the virgin state. (iii) Image sequence of the robotic fish with the virgin-state fin swimming in water. (B) (i) A 3D-printed fish fin at the strengthened state on a robotic fish. (ii) Zoom-in view of the fish fin at the strengthened state. (iii) Image sequence of the robotic fish with the strengthened-state fin swimming in water. (C) (i) A damaged fish fin installed on a robotic fish. (ii) Zoom-in view of the fish fin after being healed in water for 8 h. (iii) Image sequence of the robotic fish with the healed fin swimming in water. (D) Moving distances as functions of the swimming time for the virgin and strengthened states, and the corresponding steady velocities. (E) Moving distances as functions of the swimming time for the virgin and healed states, and the corresponding steady velocities.

The constructive adaptation in the aquatic environment can also facilitate the performance enhancement and recovery of a robotic fish with a flexible fin made from the new polymer (Fig. 5). Although the recent developments of swimming soft robots have highlighted their great potential in underwater operation and ocean exploration (24–29), their performance may still be threatened by the material degradation and damage of the employed soft polymers in the aquatic environment (30). In sharp contrast to the typically employed soft polymers, the newly proposed polymer here can constitute soft robotic systems that self-strengthen operational performance and self-heal cracks in the aquatic environment. Following the shape of the caudal fin of a commercial remotely controlled robotic fish, we employ the new polymer to 3D-print a caudal fin and then install it on the commercial robotic fish (Fig. 5A and Fig. S15). The robotic fish with the as-printed caudal fin can swim with a steady speed of 4.7 cm s^−1^ in the first trial (Fig. 5A and D and Movie S4). After the robotic fish swims in the water bath for 8 h followed by resting in the air overnight, the robotic fish can swim with a steady speed of 6.6 cm s^−1^, which increases by a factor of 140% compared to its virgin state (Fig. 5B and D and Movie S4). Note that the tail oscillating frequencies of the two swimming operations are the same (i.e. 6 Hz). The speed enhancement is because of the water-induced strengthening of the fin that provides the higher propulsive force with the same tail oscillating frequency. Next, we intentionally make fatal damage to the polymer fin by cutting a large crack in the middle [Fig. 5C(i)]. The damaged robot loses the capability of swimming in the water. After we bring the crack interfaces into contact and immerse the sample in water for 8 h (with resting in the air for overnight), the cracked interfaces can be nicely healed [Fig. 5C(ii)]. The robotic fish then resumes the capability of swimming in the water with a steady speed of around 6 cm s^−1^ (Fig. 5C and E and Movie S4), which is higher than the virgin speed (4.7 cm s^−1^) and close to the strengthen speed (6.6 cm s^−1^).

### Healable packaging polymers for flexible electronics

**Fig. 6.**
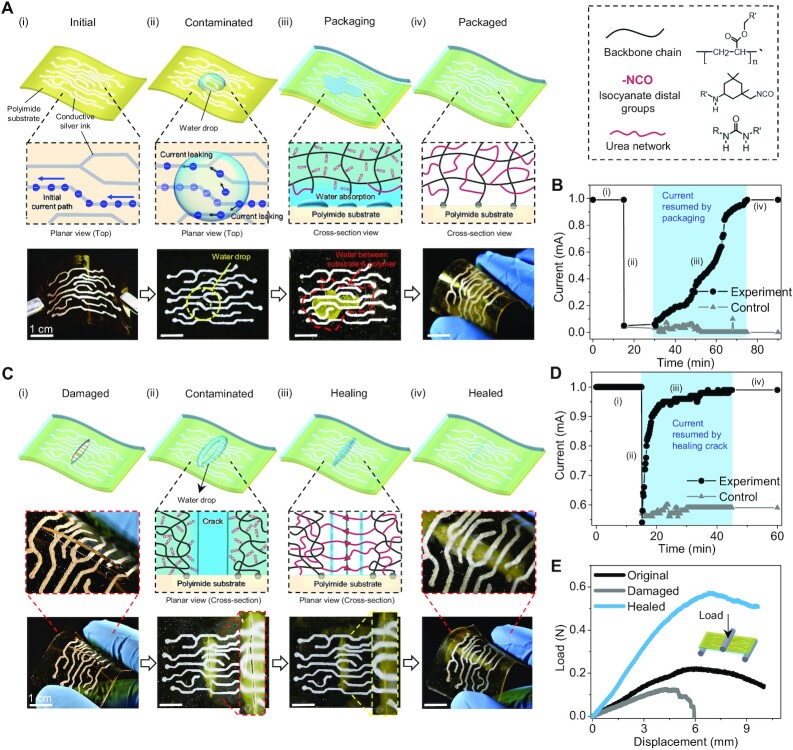
Healable packaging polymers for flexible electronics. (A) Schematics and sample images to show: (i) a flexible circuit and the corresponding conductive route, (ii) the flexible circuit contaminated by a water drop and the current being leaked from the initial conductive route, (iii) the contaminated flexible circuit being packaged with the new polymer ink, and (iv) the flexible circuit packaged with the new polymer after absorbing the water contaminant. (B) Measured current of the initial conductive route in the flexible circuit packed with the new polymer (experiment) and photocurable polyurethane (control). (C) schematics and sample images to show: (i) a crack in the packaging polymer above flexible circuit, (ii) the crack contaminated by a water drop, (iii) the crack being healed by the water drop, and (iv) the fully healed packaging polymer. (D) Measured current of the initial conductive route in the flexible circuit packed with the new polymer (experiment) and photocurable polyurethane (control). (E) Load–displacement curves of the original, damaged, and healed packaged flexible circuit under three-point–bending tests.

One of the big challenges for flexible electronics is electronic leakage during operation in wet environments (31, 32). We here present a class of novel packaging polymers that can help avoid water-induced electronic leakage of flexible electronics through reaction and healing processes. We first employ a silver ink pen to write a conductive circuit on a flexible polyimide substrate [Fig. 6A(i) and Fig. S16A], and then use a source meter to monitor the current passing one conductive route (Fig. S16B). When a water droplet (nondeionized) is contaminating the circuit, the current in the main conductive route drastically drops because of the leakage to other routes [Fig. 6A(ii) and B]. We then apply a thin layer of the new polymer ink (viscous liquid) on the contaminated circuit and employ a visible light to solidify the polymer ink to package the circuit [Fig. 6A(iii)]. During the packaging process, the water droplet is gradually absorbed by the packaging polymer through the water–NCO reaction, and the current through the initial conductive route gradually increases and resumes the initial current level [Fig. 6A(iv) and B]. The water droplet is fully absorbed over the 1-h packaging process and forms the additional cross-links within the packaging polymer matrix (Fig. 6B). As a control, we packaged the contaminated circuit using a photocurable polyurethane polymer; however, the measured current remains low over the 1-h packaging process as the electronic leakage is not stopped (Fig. 6B). In contrast to the control case, the new polymer features free NCO groups that can continuously absorb and react with water molecules and thus eliminate electronic leakage.

In addition, the proposed packaging polymer can also turn the destructive water contamination into a constructive factor to heal cracks. If the packaging polymer is cracked [Fig. 6C(i)], the external water molecules may enter the cracking region to induce electronic leakage with a sharp current drop [Fig. 6C(ii) and D]. However, the water molecules in the crack region will be gradually consumed by the packaging polymer to form interfacial chemical bridging to heal the crack [Fig. 6C(iii)], along with the recovery of the current leakage of the circuit (Fig. 6D). The healed packaging material can sustain a flexural bending deformation, without cracking the healed region [Fig. 6C(iv) and E). Compared to the virgin sample, the healed sample exhibits a larger bending stiffness (Fig. 6E and Fig. S16C), because the water molecules around the crack interface not only heal the crack but also strengthen the material by forming additional cross-links. Note that the control case with traditional polyurethane polymer as the packaging layer does not show a quick current resume because the control packaging polymer cannot harness the water to heal the crack (Fig. 6D).

## Conclusion

In summary, we report a class of 3D-printable polymers that can strengthen the material matrix and heal cracks in response to typically destructive aquatic environments. Such a constructive adaptation capability has been widely observed in biological systems (1–5, 8, 9), while rarely in traditional engineering materials that typically respond destructively to destructive environmental stressors. The key mechanism of the presented paradigm relies on chemical groups that react with the environmental stressors to form constructive cross-links, thus reversing the negative impact of the environmental stressors. This paradigm echoes the recent advance in polymer mechanochemistry, where mechanophores are employed to respond to the destructive force stressors and form constructive cross-links (3–5) or lengthen polymer strands (6). The difference of the proposed paradigm is that the employed active groups directly interact with the imposed environmental stressors without going through the mechanochemical transductions of the mechanophores (3–6); consequently, the proposed paradigm may drastically expand the existing scope that is limited to force stressors, and potentially be able to extend to other destructive environmental stressors, such as sunlight (9), greenhouse gases, high temperature, or even radiation. Additionally, we present a new class of 3D-printable photopolymers that can constructively adapt to environmental stressors. Given that the presented photopolymers may be used in various photopolymerization based 3D-printing technologies, such as stereolithography (15, 33), polyjet (34), photopolymer waveguides (35), two-photon lithography (36, 37), continuous liquid production (38), and volumetric lithography (39, 40), integrating features of constructive adaptation and complex structures may enable promising applications in artificial muscles, soft robotics, and flexible electronics, where constructive responses to environmental stressors are highly desirable.

## Materials and methods

### Materials

Poly (tetrahydrofuran) (Poly THF, average molar mass 650 g mol^−1^), isophorone diisocyanate (IPDI), dimethylacetamide (DMAc), dibutyltin dilaurate (DBTDL), 2-hydroxyethyl methacrylate (HEMA), phenylbis(2,4,6-trimethylbenzoyl)phosphine oxide (PTPO), and Sudan I were purchased from Sigma–Aldrich.

### Synthesis of polymers

The new polymer was synthesized as follows: 0.05 mol of Poly THF was preheated to evaporate moisture and oxygen for 1 h at 100°C and stirred with a magnetic stir bar. 0.1 mol of IPDI, 10 wt% of DMAc, and 1 wt% of DBTDL were added in the preheated Poly THF at 70°C,  and the mixture was stirred for 1 h. After the temperature decreased to 40°C,  0.05 mol of HEMA was added, and the mixture was stirred for another 1 h to complete the synthesis. The entire synthesis process was conducted in a nitrogen environment. The conventional polyurethane polymer was prepared following similar steps as the new polymer, except that a total of 0.1 mol HEMA was added in the last step. To prepare the 3D-printing polymer ink, 1 wt% of photoinitiator PTPO and various weight percentages of Sudan I (0% to 0.02%) were mixed with the polymer liquids and stored in a dark amber bottle until use.

### 3D-printing process

The stereolithography (SLA) 3D-printing process implemented in this paper was similar to our previous work (17, 22) (Fig. S2). The basic idea was to polymerize the synthesized resin layer by layer with prescribed thickness under a light source to form a 3D structure. To prepare the image layers of the 3D structure, a computer-aided design (CAD) model was first converted to an stereolithography (STL) file and then sliced into an image sequence. A bottom-up SLA 3D-printer was used to print the structure. The setup of the bottom-up SLA 3D-printer included a white-light projector at the bottom, an acrylic-made top-open resin box right above the projector, and a motor-controlled printing stage above the resin box. The resin box was firstly prefilled with the prepared polymer resin. The printing stage was moved down into the polymer resin, leaving a prescribed distance between the stage and the bottom of the resin box. The image of the first layer in the image sequence was then projected from the projector to the bottom of the resin box to polymerize the resin for a prescribed time. After the first layer was formed on the printing stage, the stage was lifted to a distance equal to the thickness of the next layer. In the meantime, the polymer resin refluxes to fill the space caused by stage lifting. The image of the second layer was then projected to the bottom of the resin box to polymerize the second layer. The second layer then bonds covalently with the first layer after polymerization. A 3D structure was finally formed by repeating the above-mentioned process. A Teflon membrane with low surface tension (∼20 mN m^−1^) was used to reduce the separation force between the polymerized part and the bottom of the resin box.

### Characterization of water-induced strengthening

A rectangular sample (width 10 mm, length 40 mm, and thickness 1 mm) was 3D-printed with the new polymer resin. The as-printed sample was immersed in the Deionized (DI) water for 24 h and then set in the air for 2 days. The optical transmittances of the sample at different stages were tested with a UV-vis spectrometer (Cary 60 UV-Vis spectrometer, Agilent) within the range of 380 to 720 nm. The Fourier transform infrared (FT-IR) analyses of the sample at different stages were carried out with a Spectrum TWO FT-IR Spectrometer (PerkinElmer) with a scanning range of 450 to 4000 cm^−1^ and a resolution of 0.5 cm^−1^. The uniaxially tensile stress–strain behavior of the sample was tested with a mechanical tester (model 5942, Instron) with a strain rate of 0.05 s^−1^. The fracture toughness of the sample was measured by using the pure-shear fracture test (22). Testing dimensions for the unnotched sample were 30 mm in length (${a_0}$), 5 mm in width (${L_0}$), and 1mm in thickness (${b_0}$) (Fig. S11A). For the notched samples, a notch of 15 mm was cut with a razor blade from the edge before testing. The load was applied from the two ends of the clamps to uniaxially stretch the samples at a strain rate of 0.05 ${s^{ - 1}}$. A high-speed camera was used to record the critical distance (${L_c}$) on the notched samples when the crack started to propagate. Fracture toughness energy was calculated as $U( {{L_c}} )/( {{a_0}{b_0}} )$, where $U( {{L_c}} )$ was defined as the area under stress–strain curves in the un-notched test before the corresponding critical distance in the notched test (Figs. S11B to S11E) (22).

To understand the diffusion-limited strengthening behavior, we molded cylindrical polymer samples in a plastic tube with a diameter of 5 mm and length of 20 mm. The samples were then immersed in the water and taken out for study after different periods of time. Only the central part of the sample was used for the study to eliminate the boundary effect. The cross-sections of the samples were imaged using a Canon camera (EOS 70D). The Young’s moduli of the treated samples along the longitudinal direction with various water-immersion periods were measured with the Instron mechanical tester with a strain rate of 0.05 s^−1^.

### Localized water-induced strengthening

Rectangular sample plates (15 × 15 × 1 mm) were first 3D-printed with the new polymer resin. Thin acrylic covers (thickness of 1 mm and height of 3 mm) with desired hollow patterns (i.e. wavy-pattern and circular-pattern) were cut with a laser cutter (Pro-Tech 60W ${\rm{C}}{{\rm{O}}_2}$ laser cutter) and placed on the top of the plate samples. Another cover made with EcoFlex 00–30 (Smooth-on) was then placed outside of the acrylic covers to prevent water leakage between acrylic and polymer plate (Fig. S13A). Water was then filled into the acrylic covers to allow water to penetrate the polymer plate from the top surface for 24 h, followed by resting in the air for 2 days (Fig. S13B). The stiffness map of the processed samples was measured using indentation tests with an Instron mechanical tester (Fig. S13C). A compressive force *F* is applied on the sample by a flat-end indenter with the radius of *R* = 0.5 mm with a strain rate of 0.05 s^−1^. A depth $\delta $ is applied by the cylinder indenter on the sample (Fig. S13C). The Young’s modulus is calculated as $E\,\, = \,\,F( {1 - {\nu ^2}} )/( {2R\delta } ))$, where *υ* is the Poisson’s ratio of the sample.

### Characterization of water-induced healing and bonding

For the water-induced healing experiment, a rectangular sample (20 × 3 × 2 mm) was first 3D-printed with the new polymer resin. The sample was then cut in half with a razor blade, put into contact, and immersed in different-temperature water for different periods of time. For the control experiment, the fractured sample after being put into contact was stored at room temperature without water for different periods of time. Microscopic pictures were taken with an optical microscope (Nikon Eclipse LV100ND) around the fractured interface at the fractured and healed states at a scale of 500 μm. Samples healed with various healing periods are uniaxially stretched until rupture at a strain rate of 0.05 s^−1^. The healing percentage was defined as the tensile strength of the healed sample normalized by that of the virgin sample processed with water immersion for the same periods of time. To enable sample healing at 0°C,  fractured samples were put in an ice bath sealed within a petri dish containing ice–water mixture as shown in the inset schematic in Fig. 3D.

For the water-induced interfacial bonding experiment, two rectangular samples (40 × 10 × 1 mm) were first 3D-printed with the new polymer resin. The samples were then stacked together with only half of the area being in contact (20 × 10 mm) with and without water spraying in between the contacting area. After stacking for 2 days, the interfacial bonding of the samples was measured by using the $180^\circ $ peeling test as shown in the inset schematic in Fig. 3G at a strain rate of 0.05 s^−1^.

### Constructive strengthening of robotic arms

Pneumatic robotic arms were 3D-printed with the new polymer resin and actuated using a syringe by changing the pressure of the internal air channel. The first robotic arm was first actuated to successfully lift a weight of 30 g, and then actuated in the air for 20 times (4 s cycle^−1^), followed by resting in the air for 24 h. The trained robotic arm was actuated again but failed in lifting the 30 g weight. The second robotic arm was first actuated but failed in lifting a weight of 55 g. Then, it was actuated in the water for 20 times (4 s cycle^−1^), followed by resting in water for 24 h. The trained robotic arm was actuated again to successfully lift the 55 g weight. The lifting distance of the weight was measured by the images taken with the Canon camera.

### Strengthening and healing of robotic fish fins

The fish caudal fins were 3D-printed with the new polymer resin and installed on a commercial radio remote control robotic fish (CREATE TOYS, Amazon) (Fig. S15). After the virgin robotic fish swam in the water bath (within 10 s), the fish continuously swan in the water for another 8 h, followed by resting in the air overnight. Besides, another as-printed fin was imposed by a cut with a razor blade, followed by contacting the cutting surface and immersing in water for 8 h (with resting in the air overnight) for healing the crack. During the healing process, gentle support was provided to the fin to ensure the fractured surfaces are well aligned. The moving distance and velocity of the robotic fish at various states were captured by the Canon camera.

### Healable packaging polymer for flexible circuits

The flexible circuits were fabricated with a thin polyimide substrate (thickness of 50 μm) upon which silver ink circuits were drawn using a silver ink pen (Circuit Scribe, Amazon) (Fig. S16). The silver ink has a resistance of around 1 ohm cm^−1^. The current on the circuit was powered and measured by a source meter (2,400 SourceMeter, KEITHLEY) (Fig. S16A). In the experimental case, when there was water contamination on the circuit surface, we spread a thin layer of the new polymer ink on the surface, followed by the illumination of white light for 10 min. In the control case, the new polymer ink was replaced by a photocurable polyurethane ink (16). Next, a crack was induced into the packaged layer with a razor blade for both the experimental and control cases. A water droplet was dripped on the crack area and the current change during the healing process was continuously monitored by the source meter. Three-points bending tests were conducted using the Instron mechanical tester with a strain rate of 0.05 s^−1^.

## Supplementary Material

pgac139_Supplemental_Files

## Data and Material Availability

All data are available in the manuscript or supplementary materials.

## References

[1] Coffey VG , Hawley JA 2007.; The molecular bases of training adaptation. Sports Med. 37(9):737–763. 17722947 10.2165/00007256-200737090-00001

[2] Schoenfeld BJ 2010.; The mechanisms of muscle hypertrophy and their application to resistance training. J Strength Cond Res. 24(10):2857–2872. 20847704 10.1519/JSC.0b013e3181e840f3

[3] Matsuda T , Kawakami R, Namba R, Nakajima T, Gong JP 2019.; Mechanoresponsive self-growing hydrogels inspired by muscle training. Science. 363(6426):504–508. 30705187 10.1126/science.aau9533

[4] Ramirez ALB et al. 2013.; Mechanochemical strengthening of a synthetic polymer in response to typically destructive shear forces. Nat Chem.5(9):757–761. 23965677 10.1038/nchem.1720 PMC3896090

[5] Wang Z et al. 2021.; Bio-inspired mechanically adaptive materials through vibration-induced crosslinking. Nat Mater. 20(6):869–874. 33619367 10.1038/s41563-021-00932-5

[6] Wang Z et al. 2021.; Toughening hydrogels through force-triggered chemical reactions that lengthen polymer strands. Science. 374(6564):193–196. 34618576 10.1126/science.abg2689

[7] Wu X et al. 2020.; Sunlight-Driven Biomass Photorefinery for coproduction of sustainable hydrogen and value-added biochemicals. ACS Sustain Chem Eng. 8(41):15772–15781.

[8] Popper ZA et al. 2011.; Evolution and diversity of plant cell walls: from algae to flowering plants. Annu Rev Plant Biol. 62:567–590. 21351878 10.1146/annurev-arplant-042110-103809

[9] Yu K et al. 2021.; Photosynthesis-assisted remodeling of three-dimensional printed structures. Proc Natl Acad Sci. 118(3). 10.1073/pnas.2016524118 PMC7826334 33431680

[10] Boubakri A , Haddar N, Elleuch K, Bienvenu Y 2010.; Impact of aging conditions on mechanical properties of thermoplastic polyurethane. Mater Des. 31(9):4194–4201.

[11] Schott H 1992.; Kinetics of swelling of polymers and their gels. J Pharm Sci. 81(5):467–470. 1403682 10.1002/jps.2600810516

[12] Kamata H , Akagi Y, Kayasuga-Kariya Y, Chung U-i, Sakai T 2014.. “Nonswellable” hydrogel without mechanical hysteresis. Science. 343(6173):873–875. 24558157 10.1126/science.1247811

[13] Kinloch AJ 2012.; Adhesion and adhesives: science and technology. Dordrecht: Springer Science & Business Media.

[14] Peppas NA , Buri PA 1985.; Surface, interfacial and molecular aspects of polymer bioadhesion on soft tissues. J Controlled Release. 2:257–275.

[15] Wang Q et al. 2016.; Lightweight mechanical metamaterials with tunable negative thermal expansion. Phys Rev Lett. 117(17):175901. 27824463 10.1103/PhysRevLett.117.175901

[16] Yu K et al. 2020.; Healable, memorizable, and transformable lattice structures made of stiff polymers. NPG Asia Mater. 12(1):26.

[17] Yu K , Xin A, Du H, Li Y, Wang Q 2019.; Additive manufacturing of self-healing elastomers. NPG Asia Mater. 11(1):1–11.

[18] Frisch KC , Klempner D 1998.; Advances in urethane: science & technology. Boca Raton (FL): CRC Press.

[19] Delebecq E , Pascault J-P, Boutevin B, Ganachaud F 2013.; On the versatility of urethane/urea bonds: reversibility, blocked isocyanate, and non-isocyanate polyurethane. Chem Rev. 113(1):80–118. 23082894 10.1021/cr300195n

[20] Prisacariu C 2011.; Polyurethane elastomers: from morphology to mechanical aspects. Vienna: Springer Science & Business Media.

[21] Chattopadhyay DK , Prasad PSR, Sreedhar B, Raju KVSN 2005.; The phase mixing of moisture cured polyurethane-urea during cure. Prog Org Coat. 54(4):296–304.

[22] Yu K et al. 2020.; Healable, memorizable, and transformable lattice structures made of stiff polymers. NPG Asia Mater. 12(1):1–16.

[23] Mullins L 1969.; Softening of rubber by deformation. Rubber Chem Technol. 42(1):339–362.

[24] Christianson C , Goldberg NN, Deheyn DD, Cai S, Tolley MT 2018.; Translucent soft robots driven by frameless fluid electrode dielectric elastomer actuators. Sci Robot. 3(17). 10.1126/scirobotics.aat1893 33141742

[25] Katzschmann RK , DelPreto J, MacCurdy R, Rus D 2018.; Exploration of underwater life with an acoustically controlled soft robotic fish. Sci Robot. 3(16). 10.1126/scirobotics.aar3449 33141748

[26] Aubin CA et al. 2019.; Electrolytic vascular systems for energy-dense robots. Nature. 571(7763):51–57. 31217583 10.1038/s41586-019-1313-1

[27] Ren Z , Hu W, Dong X, Sitti M 2019.; Multi-functional soft-bodied jellyfish-like swimming. Nat Commun. 10(1):1–12. 31266939 10.1038/s41467-019-10549-7 PMC6606650

[28] Zhu J et al. 2019.; Tuna robotics: a high-frequency experimental platform exploring the performance space of swimming fishes. Sci Robot. 4(34). 10.1126/scirobotics.aax4615 33137777

[29] Li G et al. 2021.; Self-powered soft robot in the Mariana Trench. Nature. 591(7848):66–71. 33658693 10.1038/s41586-020-03153-z

[30] Bilodeau RA , Kramer RK 2017.; Self-healing and damage resilience for soft robotics: a review. Front Robot AI. 4:48.

[31] Niu Y et al. 2020.; The new generation of soft and wearable electronics for health monitoring in varying environment: from normal to extreme conditions. Mater Today. 41: 219–242.,

[32] Almuslem AS , Shaikh SF, Hussain MM 2019.; Flexible and stretchable electronics for harsh-environmental applications. Adv Mater Technol. 4(9):1900145.

[33] Yu K et al. 2020.; Healable, memorizable, and transformable lattice structures made of stiff polymers. NPG Asia Mater. 12:26.

[34] Bartlett NW et al. 2015.; A 3D-printed, functionally graded soft robot powered by combustion. Science. 349(6244):161–165. 26160940 10.1126/science.aab0129

[35] Schaedler TA et al. 2011.; Ultralight metallic microlattices. Science. 334(6058):962–965. 22096194 10.1126/science.1211649

[36] Meza LR , Das S, Greer JR 2014.; Strong, lightweight, and recoverable three-dimensional ceramic nanolattices. Science. 345(6202):1322–1326. 25214624 10.1126/science.1255908

[37] Bauer J et al. 2017.; Nanolattices: an emerging class of mechanical metamaterials. Adv Mater. 29(40):1701850. 10.1002/adma.201701850 28873250

[38] Tumbleston JR et al. 2015.; Continuous liquid interface production of 3D objects. Science. 347(6228):1349–1352. 25780246 10.1126/science.aaa2397

[39] Shusteff M et al. 2017.; One-step volumetric additive manufacturing of complex polymer structures. Sci Adv. 3(12):eaao5496. 29230437 10.1126/sciadv.aao5496 PMC5724355

[40] Kelly BE et al. 2019.; Volumetric additive manufacturing via tomographic reconstruction. Science. 363(6431):1075–1079. 30705152 10.1126/science.aau7114

