# Supplementary material for: Constructive adaptation of 3D-printable polymers in response to typically destructive aquatic environments
Source: PNAS Nexus. 2022 Jul 29;1(3):pgac139. doi: 10.1093/pnasnexus/pgac139 (PMC9896903; doi:10.1093/pnasnexus/pgac139)
Supplement: pgac139_Supplemental_Files [file pgac139_supplemental_files.zip › PNASNEXUS-PNASNEXUS-2022-00088-s01.docx]

**Supplementary Information for**

**Constructive adaptation of 3D-printable polymers in response to the typically destructive aquatic environment**

Kunhao Yu^1^, Zhangzhengrong Feng^1^, Kyung Hoon Lee^1^, Ketian Li^1^, Haixu Du^1^, Yanchu Zhang^1^, Qiming Wang^1*^

^1^Sonny Astani Department of Civil and Environmental Engineering, University of Southern California, Los Angeles, California 90089, USA

^*^Correspondence to: qimingw@usc.edu (Q. Wang)

**This PDF file includes:**

Figs. S1 to S16
Captions for Movies S1 to S4
References

**Other Supplementary Information for this manuscript:**

Movies S1 to S4

**
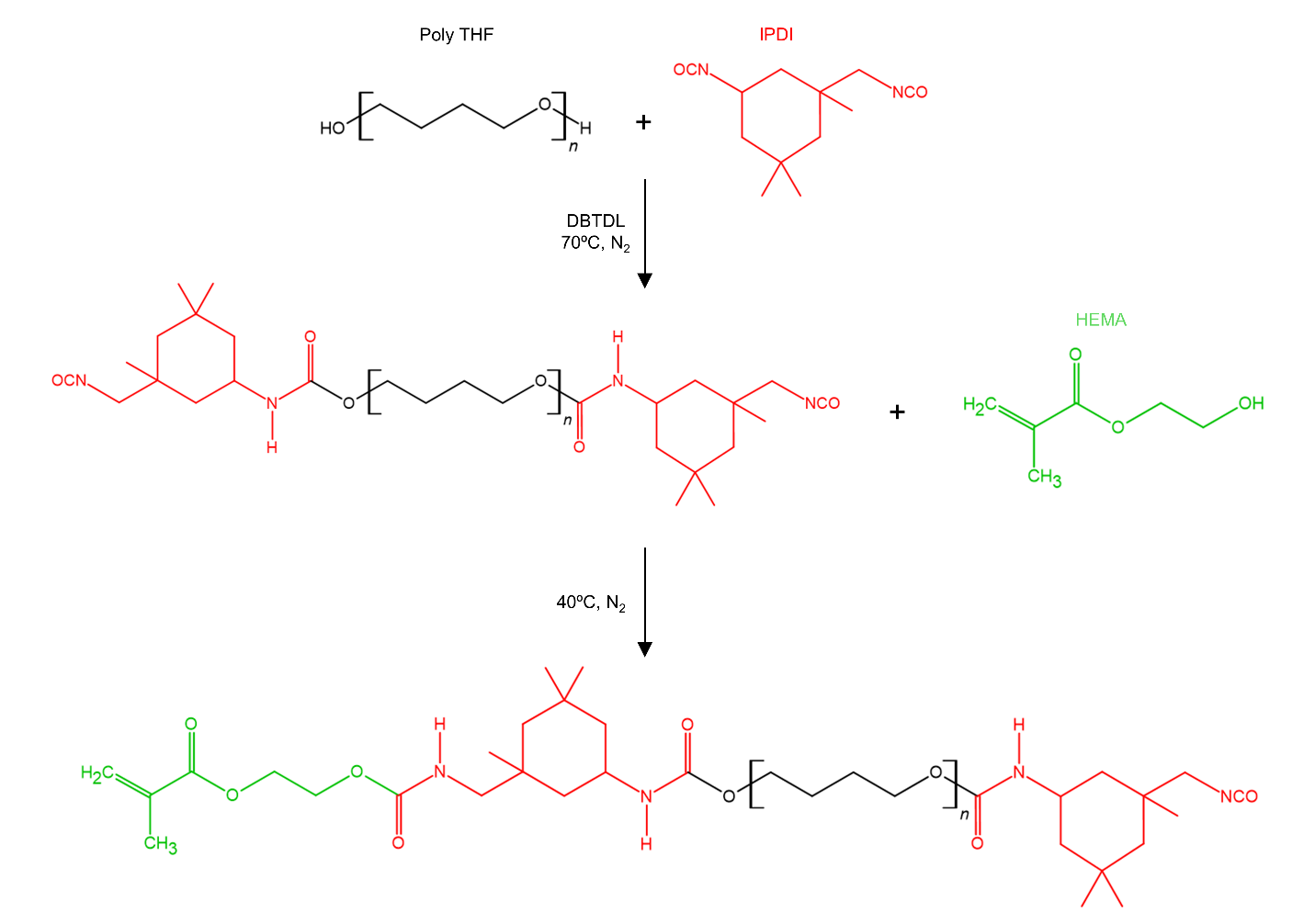
** **Fig. S1. Synthesis of the new polymer ink.** 0.05 mole of Poly THF was first preheated to evaporate moisture and oxygen at 100°C for 1 h and the mixture was stirred with a magnetic stir bar. 0.1 mole of IPDI, 10 wt% of DMAc, and 1 wt% of DBTDL were added in the preheated Poly THF at 70°C, and the mixture was mixed for 1 h. After the temperature declined to 40°C, 0.05 mole of HEMA was added, and stirred for another 1 h to complete the synthesis. The entire synthesis process was conducted in a Nitrogen environment.


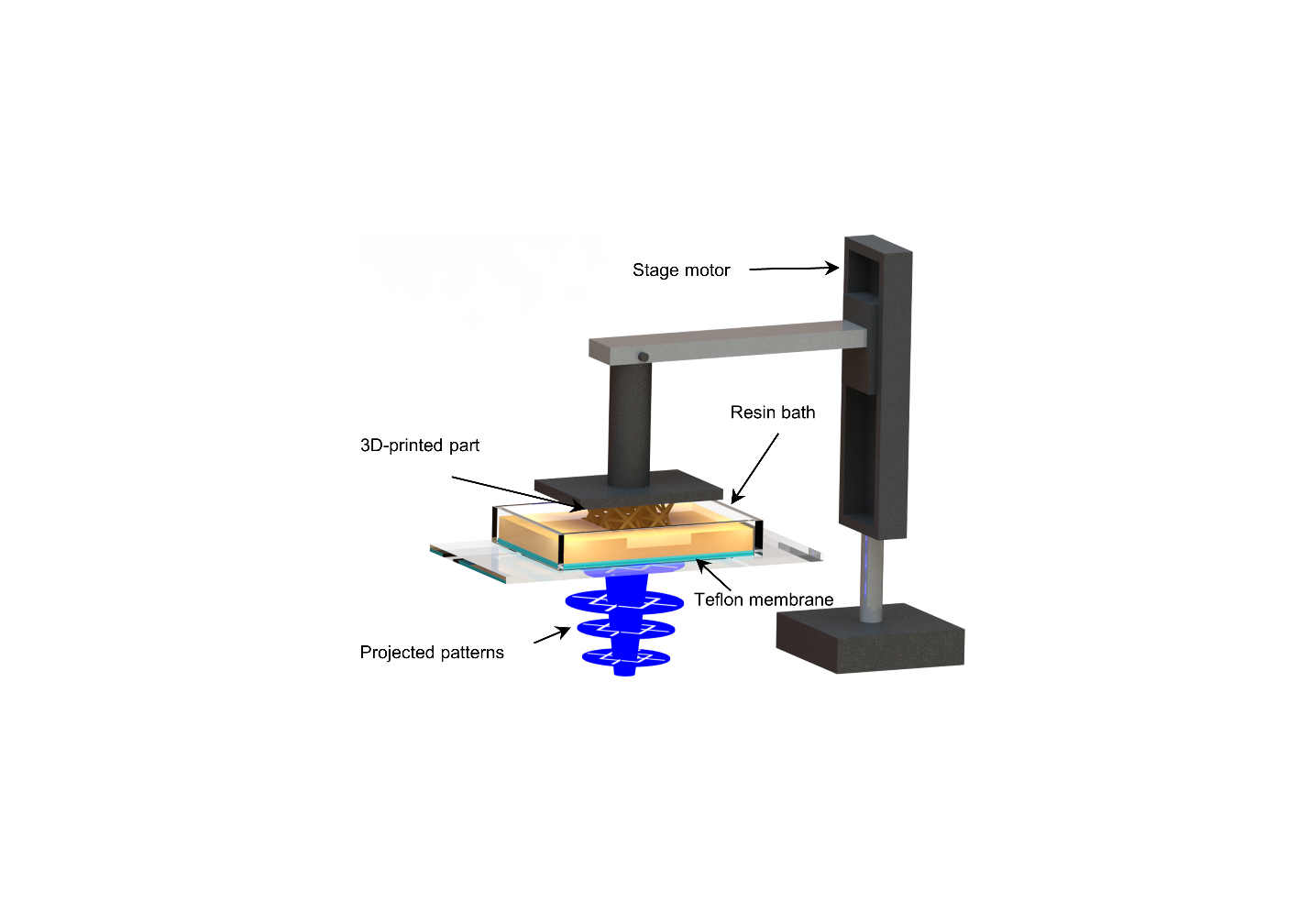


**Fig. S2. Stereolithography (SLA) 3D printing process.** The setup of the bottom-up SLA 3D printer includes a white-light projector at the bottom, an acrylic-made top-open resin box right above the projector, and a motor-controlled printing stage above the resin box. The resin box was firstly pre-filled with the prepared polymer resin. The printing stage was moved down into the polymer resin, leaving a prescribed distance between the stage and the bottom of the resin box. The image of the first layer in the image sequence was then projected from the projector to the bottom of the resin box to polymerize the resin for a prescribed time. After the first layer was formed on the printing stage, the stage was lifted to a distance of the thickness of the next layer. In the meantime, the polymer resin refluxes to fill the space caused by stage lifting. The image of the second layer was then projected to the bottom of the resin box to polymerize the second layer. The second layer then bonds covalently with the first layer after polymerization. A 3D structure was finally formed by repeating the above-mentioned process. A Teflon membrane with low surface tension (~20 mN/m) was used to reduce the separation force between the polymerized part and the bottom of the resin box.


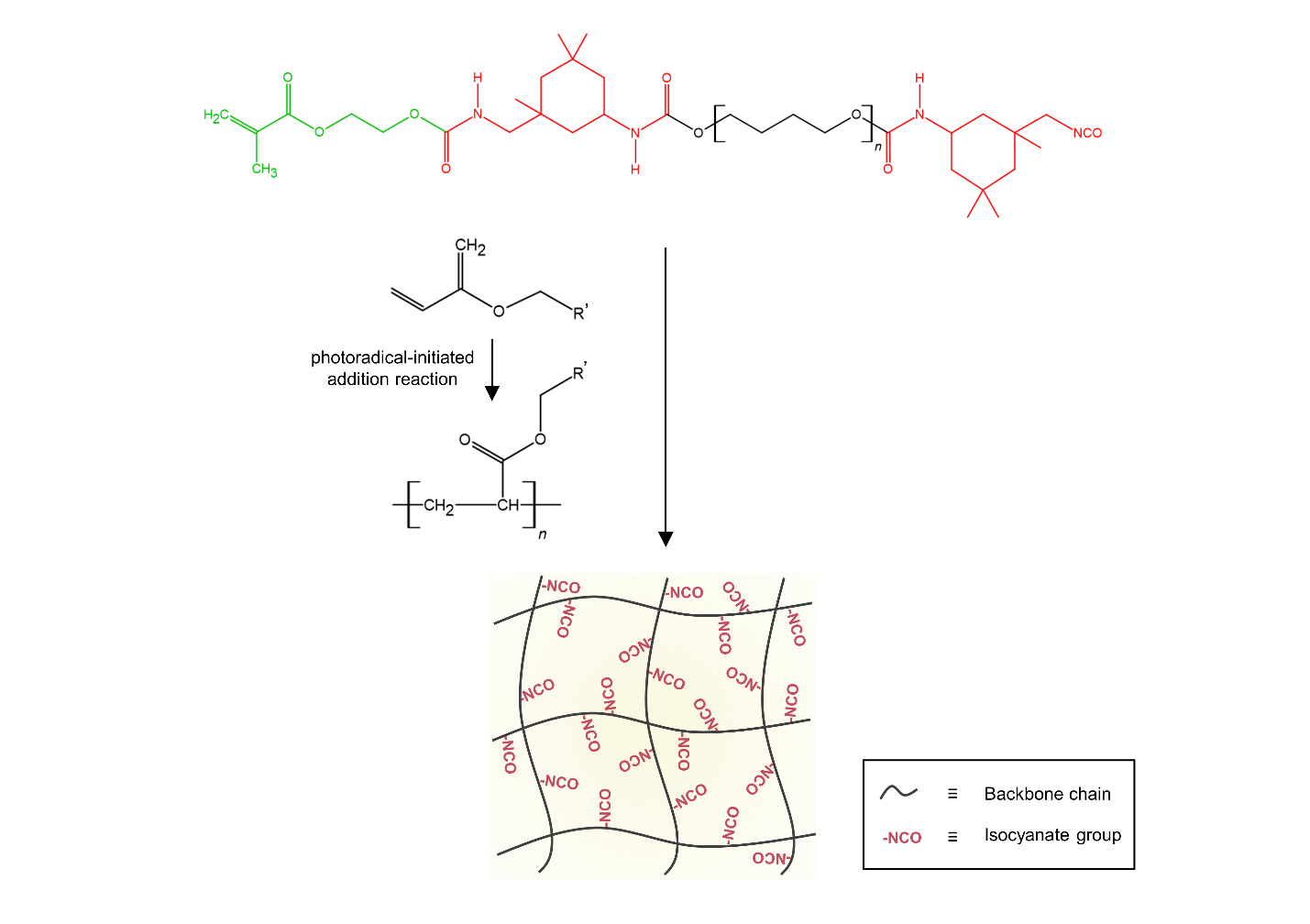


**Fig. S3. Photopolymerization of polymer ink.** The polymer resin with 1 wt% of photoinitiator (phenylbis(2,4,6-trimethylbenzoyl)phosphine oxide) and various weight percentages of Sudan I (0%-0.02%) went through a photoradical-initiated addition reaction with the photopolymerization-based stereolithography system to photopolymerize the polymer resin.

**
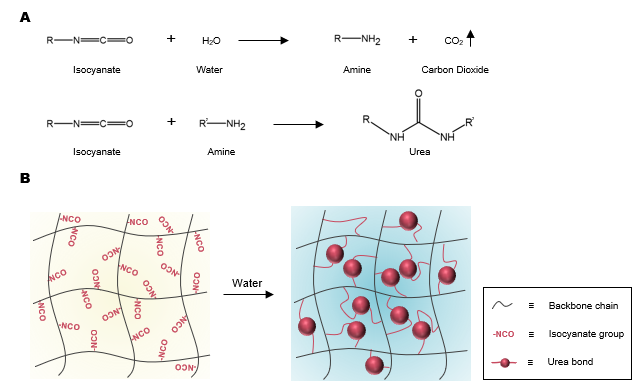
**

**Fig. S4. The chemical reaction between isocyanate groups and water molecules.** (A) Chemical schemes to show the reaction between one water molecule and two NCO groups. (B) Schematic to illustrate the water-induced chemical bridging to form additional crosslinks.


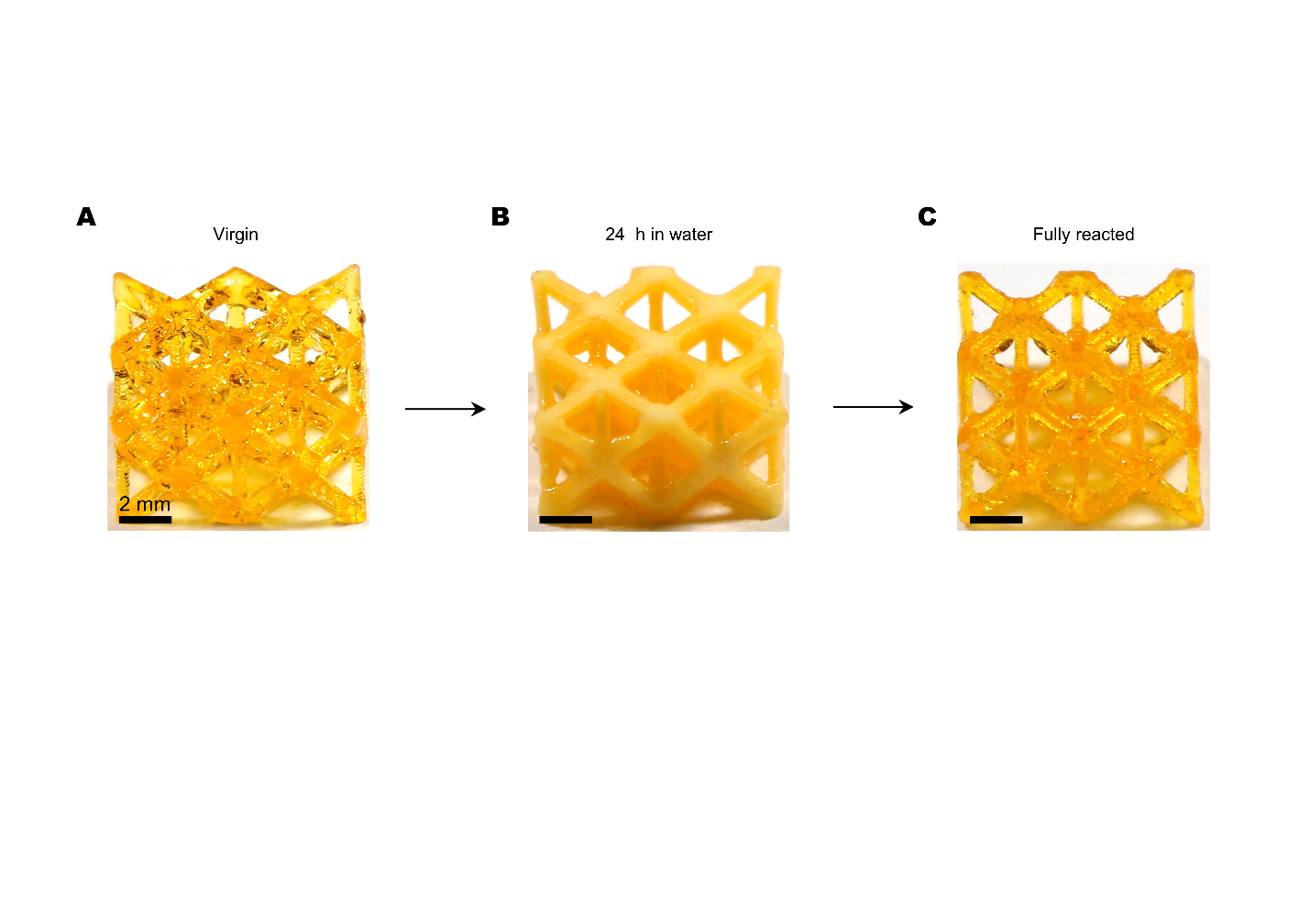


**Fig. S5. Water-assisted strengthening of a 3D-printed lattice structure.** (A) As-printed lattice structure. (B) lattice structure after being immersed in the water for 24 h. (C) Fully-reacted lattice structure after resting in the air for 2 days.


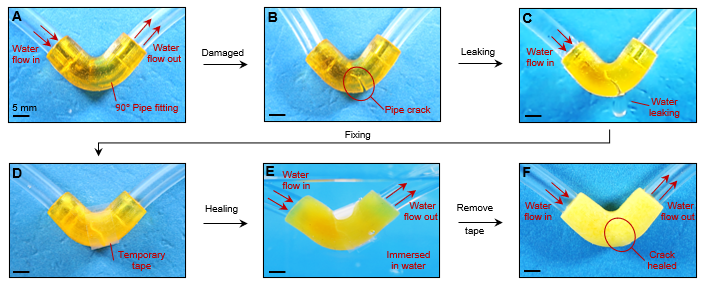


**Fig. S6. Water-assisted healing of a 3D-printed pipe fitting.** (A) A 90$^{\circ}$ pipe fitting with a water flow. (B) A crack installed on the pipe fitting. (C) Water leaks out of the pipe fitting when there is a water flow. (D) A temporary tape wrapped around to contact the crack surface of the damaged pipe fitting. (E) The wrapped pipe fitting immersed in water along with the inner water flow. (F) The healed pipe fitting sustaining a water flow without leaking.


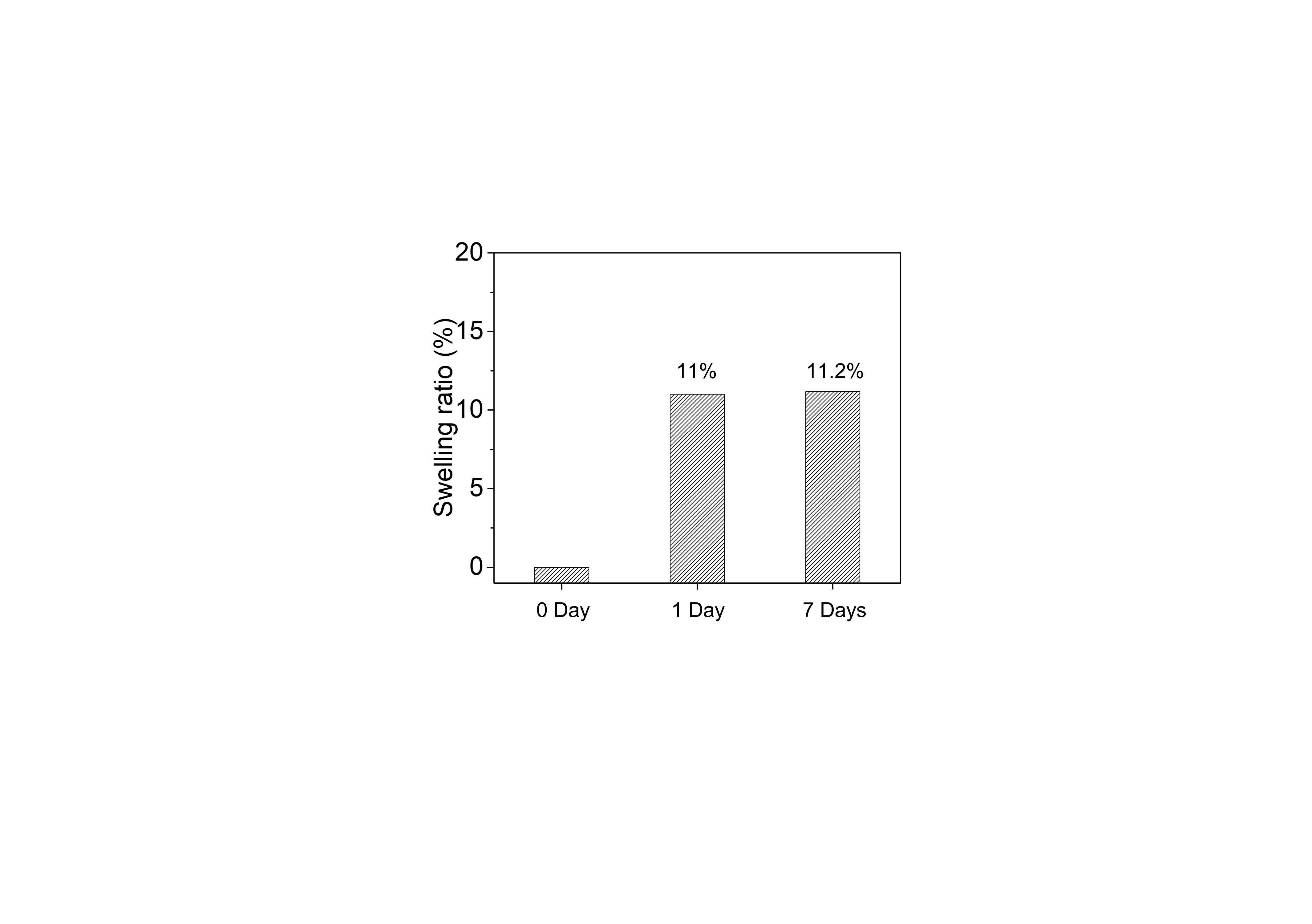


**Fig. S7. The swelling ratio of a strip sample after being immersed in water for different time periods.** The swelling ratio is calculated as the weight change of the sample after being immersed in water divided by the initial weight of the sample.


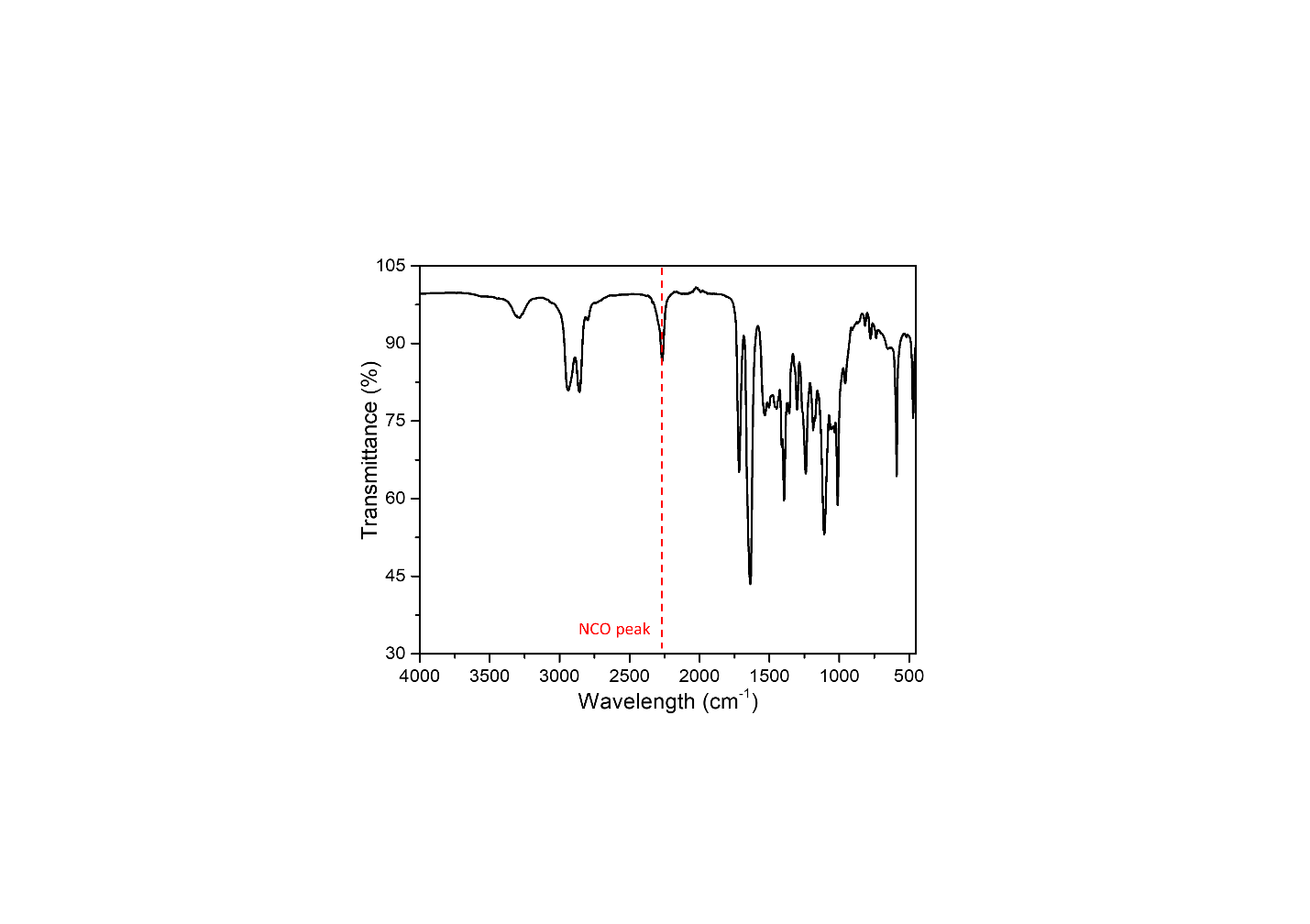


**Fig. S8. Full FTIR spectra of the new polymer sample at virgin state.** A distinct peak centered at 2,270 $cm^{-1}$was observed to indicate the existence of the NCO group.


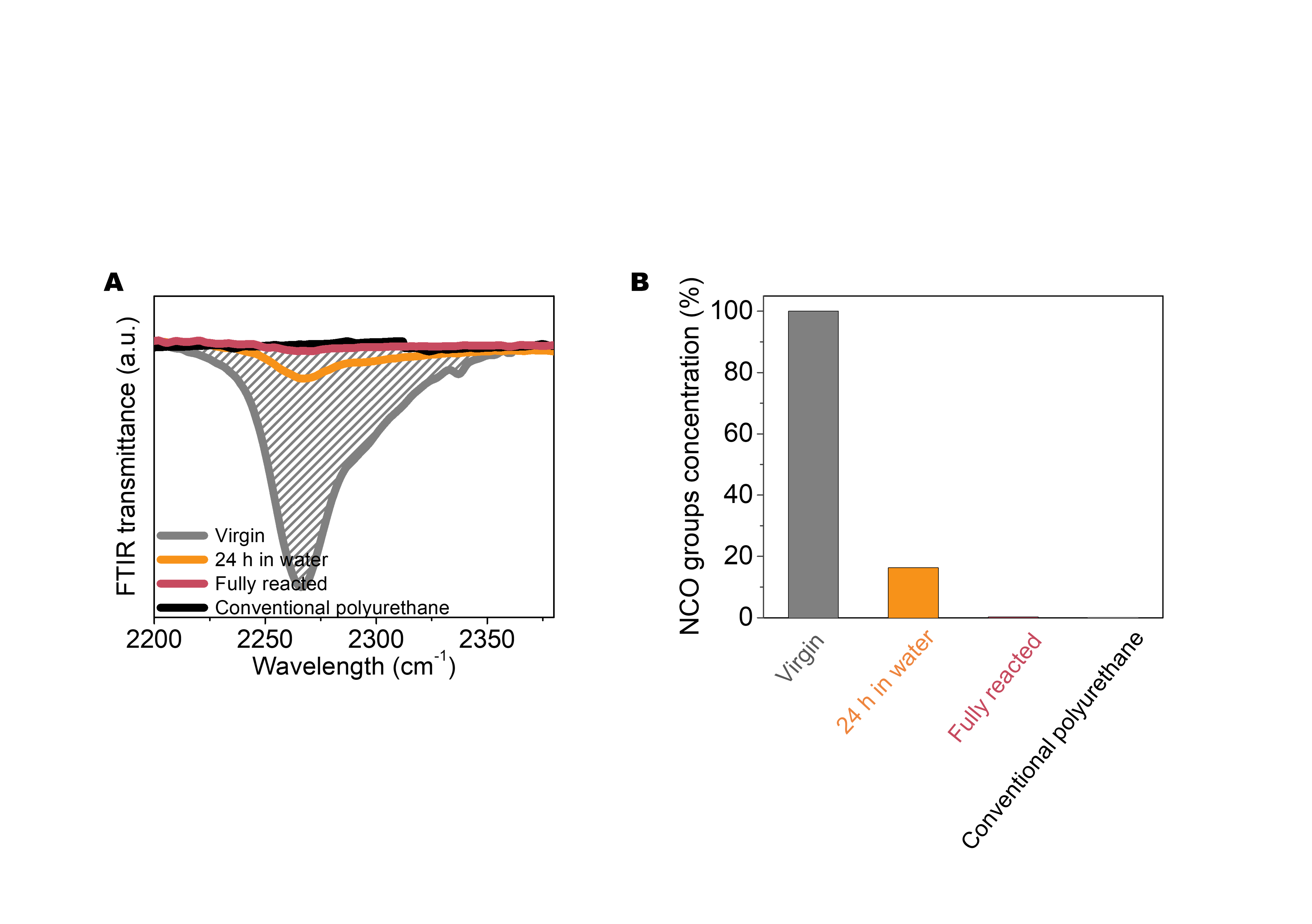


**Fig. S9.** (a) FTIR spectra within the range of 2200 $cm^{-1}$ to 2375 $cm^{-1}$ of the samples at different states and a conventional polyurethane polymer. The shaded region is the peak area to calculate the concentration of the NCO groups at the virgin state ($S_{0}$). (b) The NCO group concentration at different states and a conventional polyurethane polymer. The NCO group concentration is calculated as the area (𝑆) under the peak at 2270 $cm^{-1}$ normalized by the area at the virgin state ($S_{0}$).


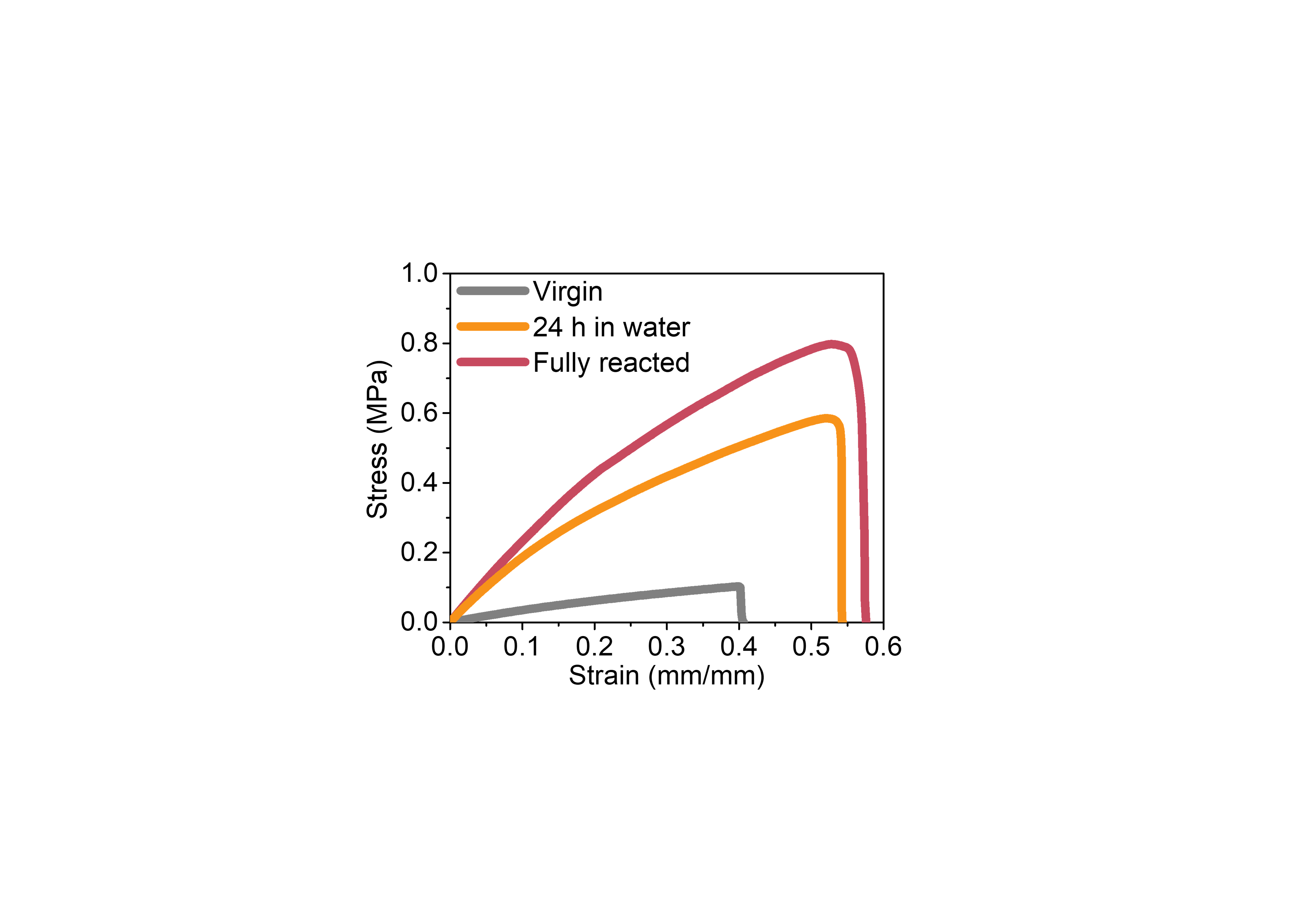


**Fig. S10. Uniaxial tensile stress-strain curves of the sample at the virgin state, after being immersed in water for 24 h, and at the fully-reacted state.**


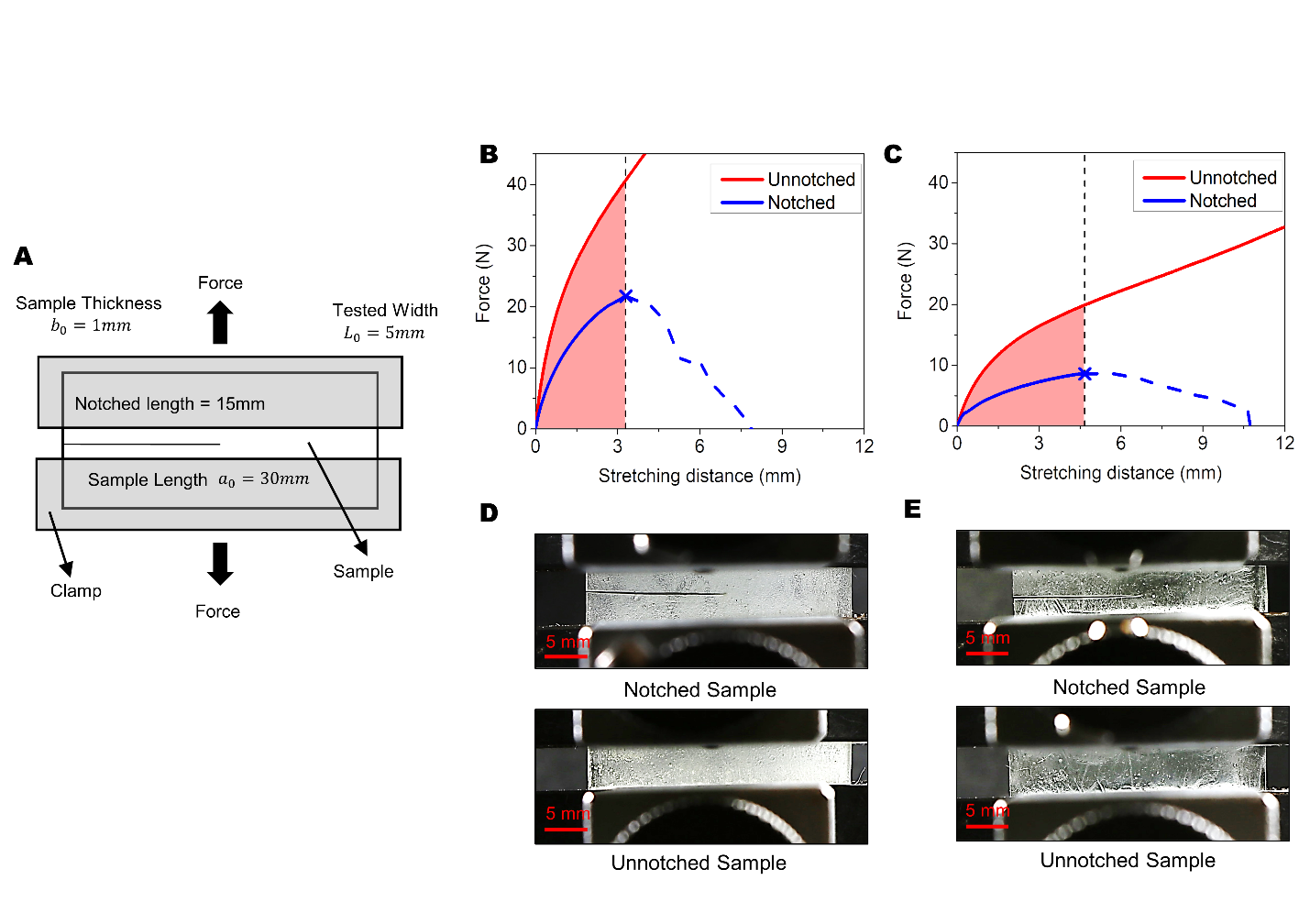


**Fig. S11. Fracture tests of the polymer samples.** (A) Sample geometry of pure-shear fracture toughness tests. (B) Force-distance curves of notched and unnotched samples at the fully-reacted state. (C) Force-distance curves of notched and unnotched samples at the virgin state. (D) Experiment images of notched and unnotched samples at fully-reacted state. (E) Experiment images of notched and unnotched samples at the virgin state.


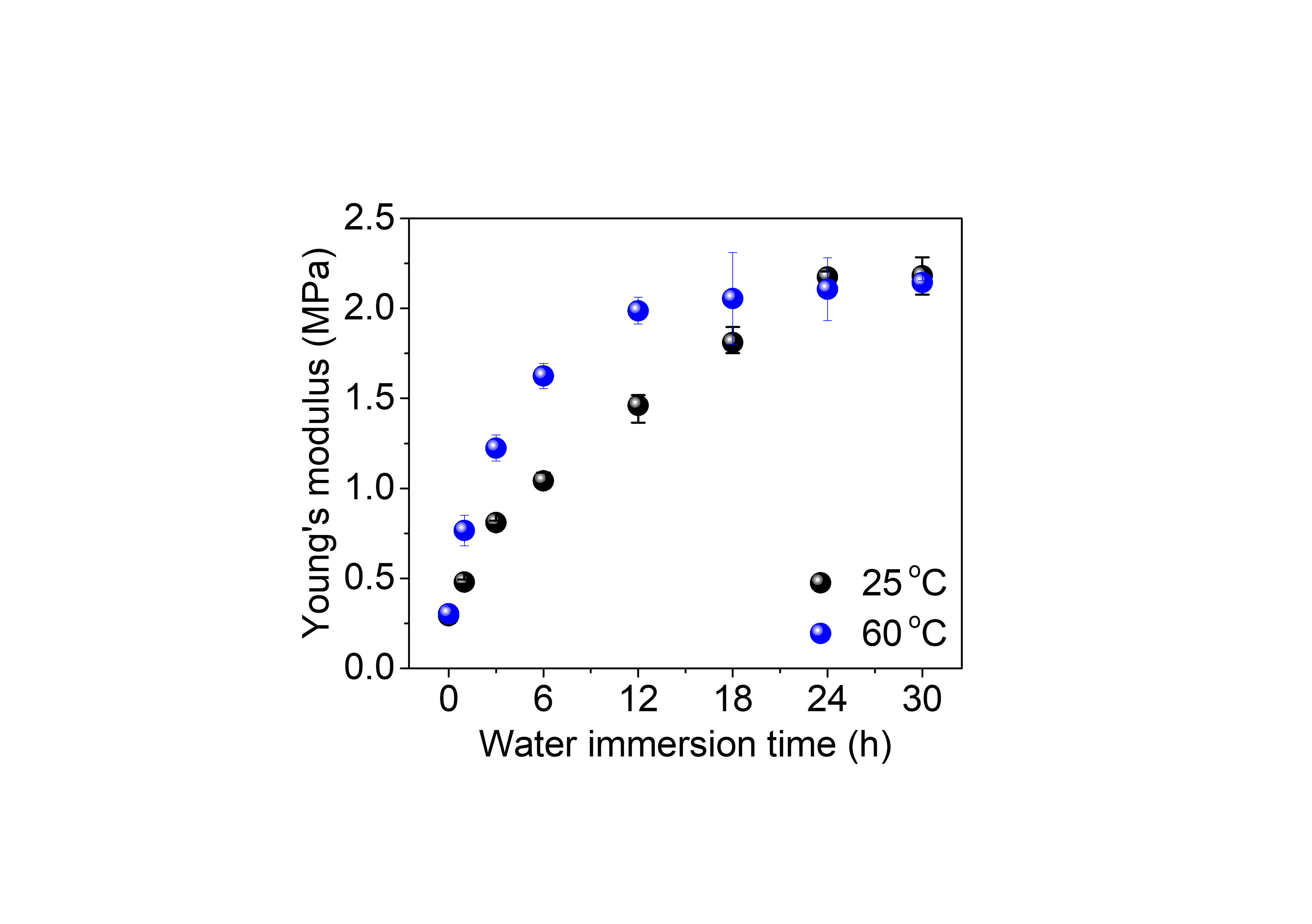


**Fig. S12. The longitudinal Young’s modulus of the cylindrical sample (Fig. 2H) as a function of the water-immersion time at 25**$\boldsymbol{℃}$ **and 60**$\boldsymbol{℃}$**.**


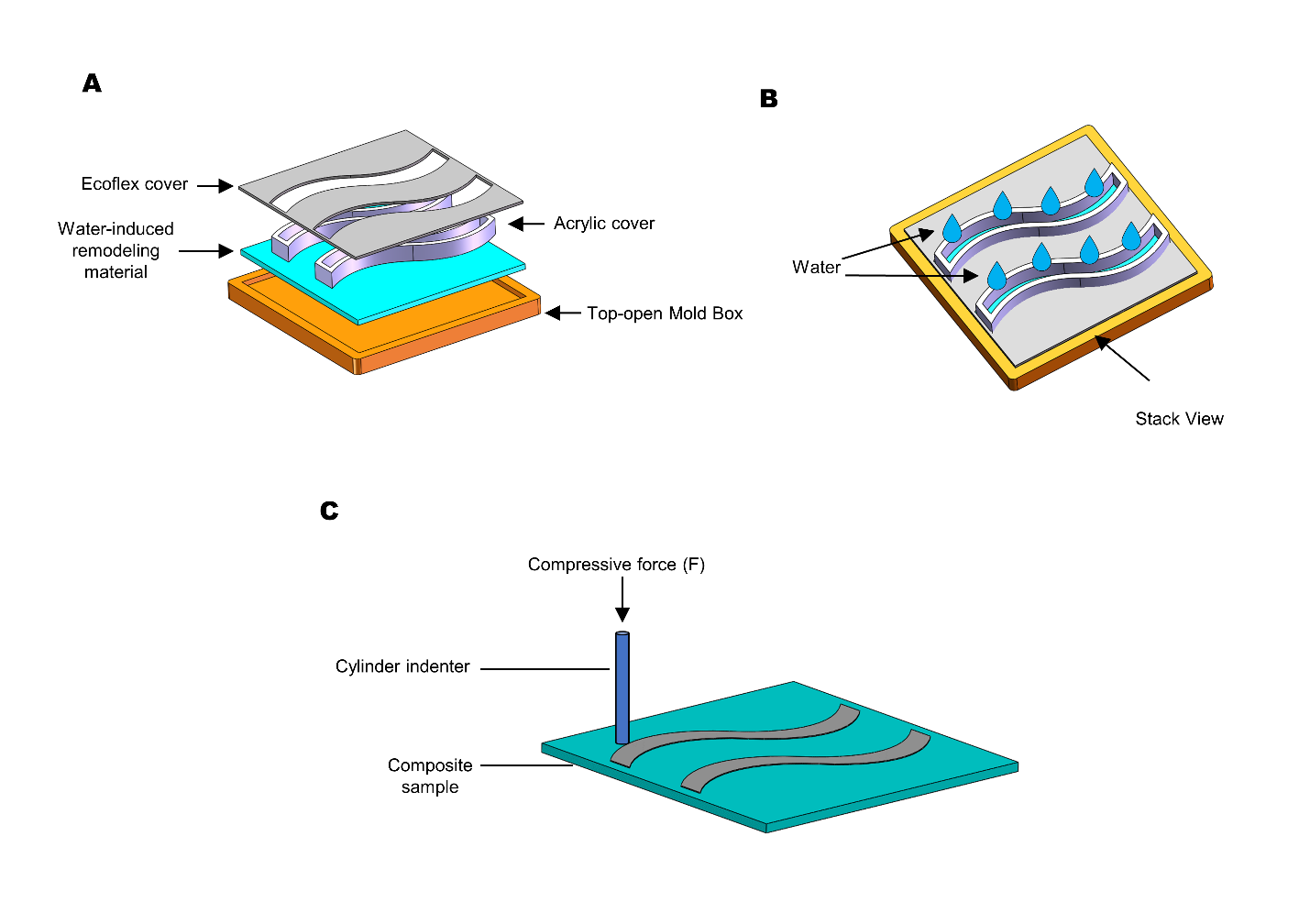


**Fig. S13. Experiments for the water-induced local strengthening.** (A) Rectangular sample plates (35 mm x 35 mm x 1 mm) were first 3D-printed with the synthesized polymer resin. Thin acrylic covers (thickness of 1 mm and height of 3 mm) with desired hollow patterns (i.e., wavy-pattern) were cut with a laser cutter (Pro-Tech 60W $CO_{2}$ Laser Cutter) and placed on the top of the plate samples. Another cover made with EcoFlex 00-30 (Smooth-on) was then placed outside of the acrylic covers to prevent water leakage between the acrylic and polymer plate. (B) Water was then filled into the acrylic covers to allow water to penetrate the polymer plate from the top surface for 24 h, followed by resting in the air for 2 days. (C) Indentation test to measure the stiffness map of the processed sample. A compressive force F is applied on the sample by a flat-end indenter with the radius of R=1 mm with a strain rate of 0.05 s^-1^. A depth $\delta$ is created by the cylinder indenter on the sample. The Young’s modulus is calculated as $E=F(1-\nu^{2})/(2R\delta))$, where υ is the Poisson’s ratio of the sample.


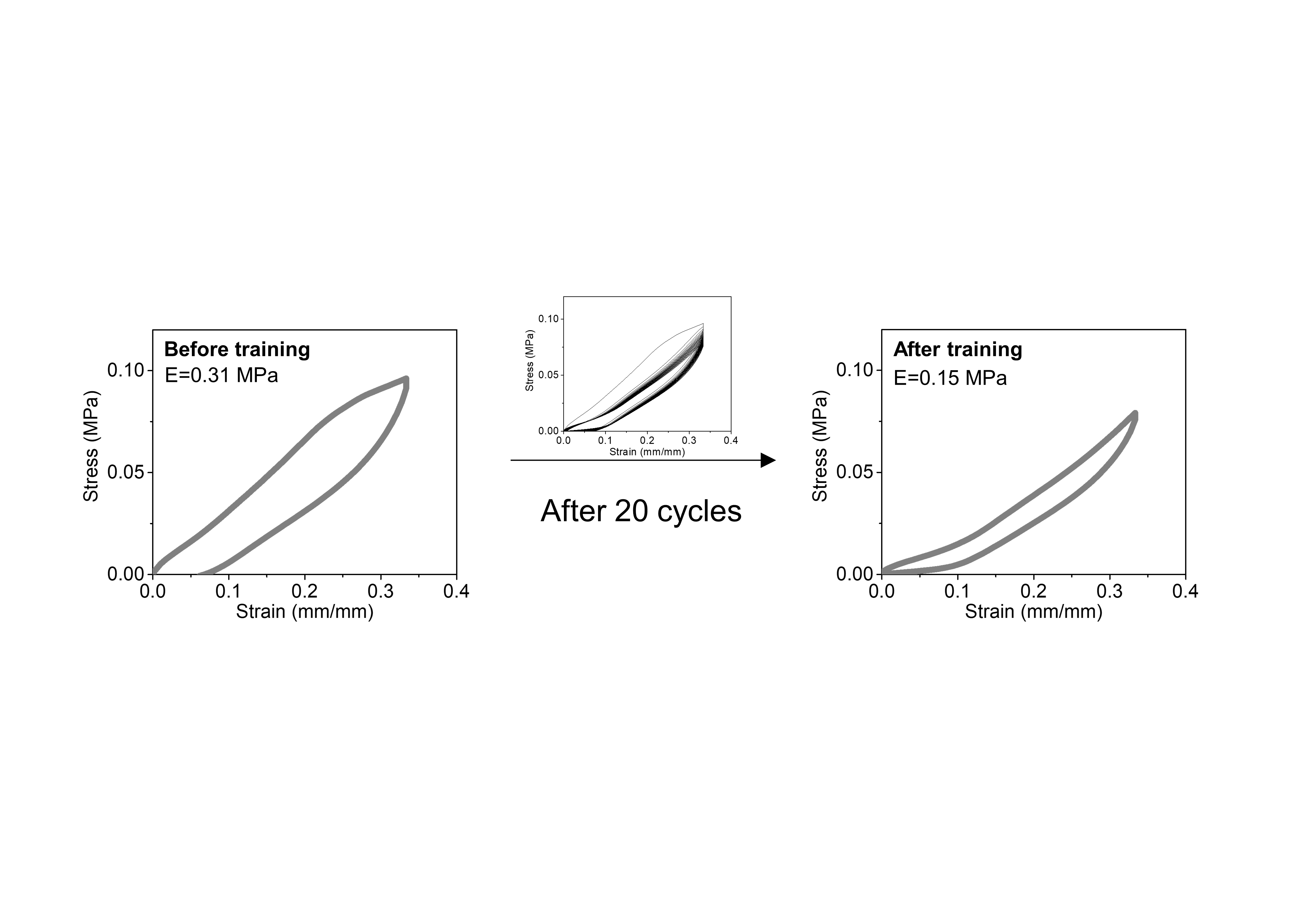


**Fig. S14. The stress-strain behavior of the as-printed material sample before and after 20 cyclic loadings.** The Young’s modulus degrades by half after 20-cycle loading.

**
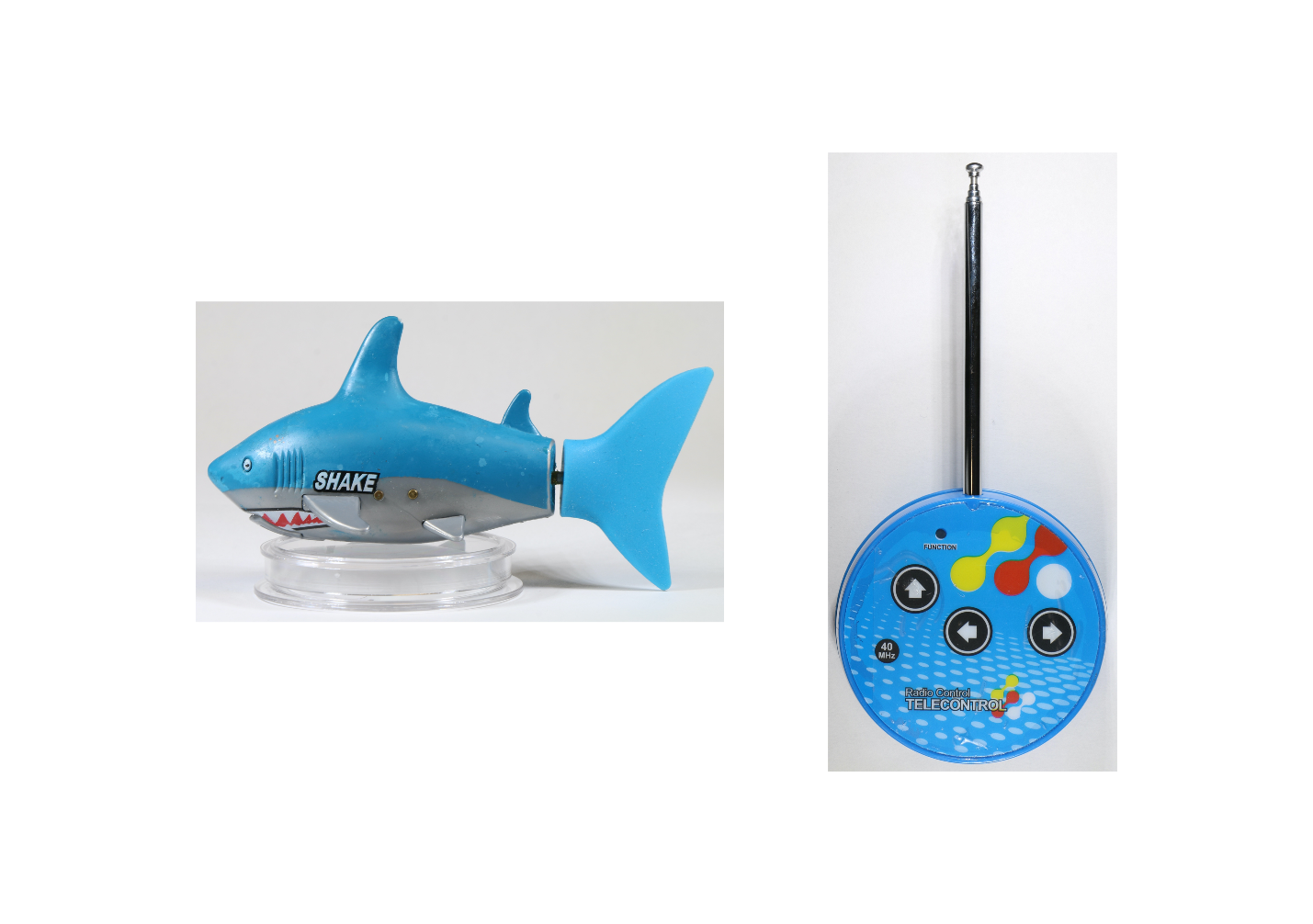
**

**Fig. S15.** A commercial remotely-controlled robotic fish with a rubber caudal fin and the telecontroller.

**
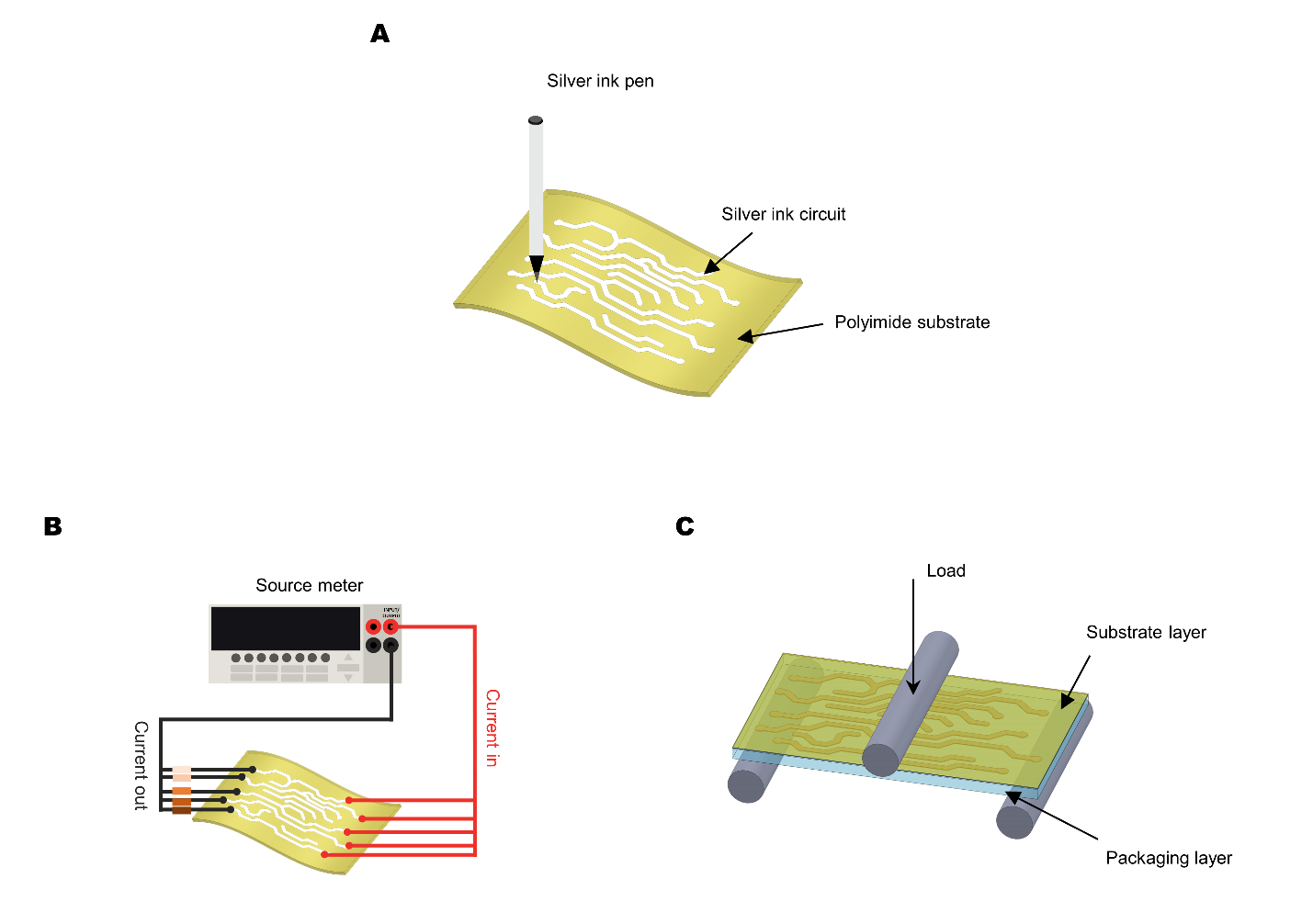
**

**Fig. S16.** (A) The fabrication process of a flexible circuit with a silver conductive ink pen (B) Schematic of the flexible circuit connected to a source meter (C) Schematic of the packaged circuit under three-points bending test.

## Captions for movies

**Movie S1.**

A movie to show that a damaged 3D-printed pipe fitting with water leaking through the crack and that water-induced healing of a 3D-printed pipe fitting resumes the water flow without leaking.

**Movie S2.**

A movie to show that an as-printed pneumatic robotic arm succeeds in lifting a weight of 30 g before training and fails in lifting the 30-g weight after training in the air for 20 cycles (along with resting in the air for 24 h).

**Movie S3.**

A movie to show that an as-printed pneumatic robotic arm fails in lifting a weight of 55 g before training and succeeds in lifting the 55-g weight after training in the water for 20 cycles (along with resting in the water for 24 h).

**Movie S4.**

A movie to show a robotic fish swim in water for 4 s with a virgin caudal fin, a strengthened caudal fin, and a water-healed caudal fin.
